# IRS4, a novel modulator of BMP/Smad and Akt signalling during early muscle differentiation

**DOI:** 10.1038/s41598-017-08676-6

**Published:** 2017-08-18

**Authors:** Gina Dörpholz, Arunima Murgai, Jerome Jatzlau, Daniel Horbelt, Mohammad Poorgholi Belverdi, Christina Heroven, Isabelle Schreiber, Gisela Wendel, Karen Ruschke, Sigmar Stricker, Petra Knaus

**Affiliations:** 1 Institute for Chemistry and Biochemistry, Freie Universität Berlin, Thielallee 63, 14195 Berlin, Germany; 2 Berlin-Brandenburg School for Regenerative Therapies, Charité Universitätsmedizin Berlin, Campus Virchow-Klinikum, Augustenburger Platz 1, 13353 Berlin, Germany; 3 Max Planck Institute for Molecular Genetics, Ihnestraße 63-73, 14195 Berlin, Germany

## Abstract

Elaborate regulatory networks of the Bone Morphogenetic Protein (BMP) pathways ensure precise signalling outcome during cell differentiation and tissue homeostasis. Here, we identified IRS4 as a novel regulator of BMP signal transduction and provide molecular insights how it integrates into the signalling pathway. We found that IRS4 interacts with the BMP receptor BMPRII and specifically targets Smad1 for proteasomal degradation consequently leading to repressed BMP/Smad signalling in C2C12 myoblasts while concomitantly activating the PI3K/Akt axis. IRS4 is present in human and primary mouse myoblasts, the expression increases during myogenic differentiation but is downregulated upon final commitment coinciding with Myogenin expression. Functionally, IRS4 promotes myogenesis in C2C12 cells, while IRS4 knockdown inhibits differentiation of myoblasts. We propose that IRS4 is particularly critical in the myoblast stage to serve as a molecular switch between BMP/Smad and Akt signalling and to thereby control cell commitment. These findings provide profound understanding of the role of BMP signalling in early myogenic differentiation and open new ways for targeting the BMP pathway in muscle regeneration.

## Introduction

Cellular growth and differentiation are regulated by a multitude of distinct signalling pathways. Crosstalk between these pathways is indispensable to ensure a balanced adaptation to certain signalling inputs thereby facilitating specificity of signalling responses.

The Insulin Receptor Substrate 4 (IRS4) belongs to the Insulin Receptor Substrate (IRS) family of scaffold proteins and provides docking sites for various signalling proteins^1^. Similar to other IRS family members, IRS4 was reported to associate with phosphatidylinositol-3-kinase (PI3K) and Growth factor receptor-bound protein 2 (Grb2)^2^, to mediate GLUT4 translocation^3^ and to regulate cell proliferation^4–6^. There is, however, increasing evidence that IRS4 displays distinct signalling features since it does not interact with either SHP-2 or phospholipase C_γ_^2^ nor does it trigger cell survival in myeloid progenitor cells^7^. Some studies even suggest a role for IRS4 in suppressing the function of other IRS proteins in IGF1-mediated signalling^8^. Moreover, IRS4 was reported to be no substrate for the insulin receptor in muscle tissue^9^. In contrast to IRS1/2, IRS4 is expressed in a tissue-specific manner, predominantly in brain, kidney and skeletal muscle^4, 9, 10^. This could explain why mice lacking IRS4 show only mild defects in growth and glucose homeostasis^11^. Taken together, the physiological function and relevance of IRS4 still remain elusive.

Bone Morphogenetic Proteins (BMPs) are pleiotropic cytokines belonging to the Transforming Growth Factor-β (TGF-β) superfamily. They fulfil various cellular functions both during embryonic development and in adult tissue homeostasis by regulating distinct processes in a context-specific manner^12, 13^. BMPs signal via binding to heteromeric complexes of two types of transmembrane serine/threonine kinase receptors, the BMP type I (ACVRI, BMPRIA, BMPRIB) and type II receptors (ActRIIa, ActRIIb, BMPRII). Upon ligand binding the activated type I receptor kinase phosphorylates cytosolic receptor-regulated Smads1/5/8 (R-Smads). This in turn induces their oligomerisation with the common-mediator Smad4 (co-Smad) followed by subsequent nuclear translocation and transcriptional regulation of specific BMP/Smad target genes like *inhibitors of differentiation* (IDs)^14, 15^. Besides the canonical Smad pathway, BMPs induce non-Smad signalling like Mitogen-activated protein kinases (MAPK) such as p38, JNK and ERK but also PI3K/Akt-mediated routes^16^. Since malfunction of BMP signalling is intimately linked with severe diseases including cardiovascular and musculoskeletal disorders, cancer and fibrosis^17^, tight regulation and fine-tuning are indispensable. This may occur at multiple levels of the signalling cascade for instance by extracellular antagonists, co-receptors or by receptor internalisation^18^. Another layer of regulation is achieved by cytosolic proteins binding to the BMP receptors like LIM kinase 1 (LIMK1) or cyclic guanosine-monophosphate (cGMP)-dependent kinase I (cGKI)^19, 20^. A﻿lso the BMP pathway crosstalks to other signalling pathways, e.g. Hippo and MAPK^21–23^ thereby regulating cell growth and differentiation.

Here, we present IRS4 as a novel player in the BMP pathway, which physically interacts with the BMP receptor BMPRII and affects BMP-induced signalling in myoblasts. We show that IRS4 interferes with BMP signal transduction by impinging on the abundance of its downstream signalling component Smad1. The IRS4-dependent decrease of Smad1 protein is linked to enhanced ubiquitination and subsequent degradation of Smad1 resulting in its reduced transcriptional activity. In addition, IRS4 affects the non-Smad signalling branch by promoting Akt signalling in muscle cells. Furthermore, our data provide clear evidence that IRS4 is expressed in myoblasts during mouse limb development as well as in postnatal satellite cells suggesting its involvement in myogenesis. We show that the BMPRII interacting IRS4 serves as a novel platform for inhibition of BMP/Smad signalling along with an activation of the PI3K/Akt axis to promote differentiation of precursor cells to the myogenic lineage.

## Results

### IRS4 interacts with BMPRII in a ligand-independent manner at the plasma membrane

**Figure 1:**
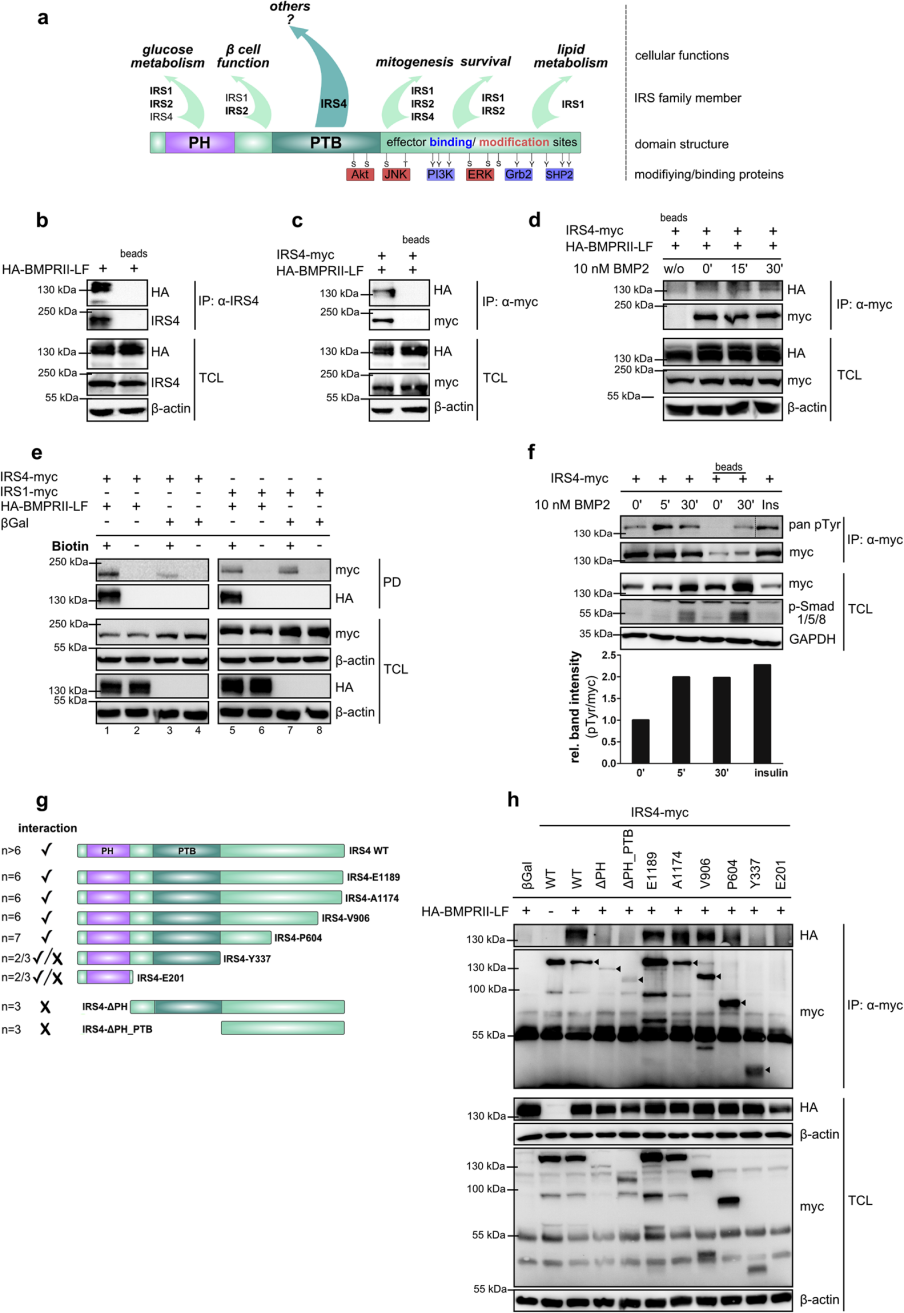
IRS4 interacts with BMPRII-LF in a ligand independent manner at the plasma membrane. (**a**) Scheme depicting the IRS protein domain structure, prominent interacting as well as modifying proteins and so far described cellular functions; the main IRSs for the respective functions are represented in bold letters. PH = pleckstrin homology domain; PTB = phosphotyrosine binding domain (**b**,**c**) IRS4 interacts with BMPRII-LF. Transfected HEK293T cells were subjected to immunoprecipitation using α-IRS4 (left) and α-myc (right) antibodies. Immunoprecipitates (IP) and total cell lysate (TCL) were analysed by Western blotting using indicated antibodies. Incubation with beads only served as IP control. (**d**) The IRS4/BMPRII interaction is ligand-independent. Transfected HEK293T cells were stimulated with 10 nM BMP2 for indicated times. IRS4-myc was immunoprecipitated; precipitates and TCL were analysed by Western blotting using indicated antibodies. Incubation with beads only served as IP control. (**e**) IRS4 interacts with cell surface exposed BMPRII-LF. Transfected HEK293T cells were incubated with biotin solution to label surface receptors followed by pull-down using streptavidin coupled beads. Precipitates and TCL were subjected to Western blotting using indicated antibodies. (**f**) IRS4 is tyrosine phosphorylated upon BMP2 stimulation. Transfected HEK293T cells were stimulated with 10 nM BMP2 for indicated times; 100 nM insulin (Ins) served as positive control. IRS4-myc was immunoprecipitated; precipitates and TCL were analysed by Western blotting. Quantification depicts pan pY signals normalised to precipitated IRS4-myc. Incubation with beads only served as IP control. (**g**) Scheme depicting IRS4 WT and truncated mutants thereof. Interaction (✓) or no interaction (x) is indicated on the left side; n refers to the number of performed experiments. (**h**) Mapping of the BMPRII binding region at IRS4. Transfected HEK293T cells were subjected to immunoprecipitation using an α-myc antibody. The expected molecular weight of IRS4 wild type (WT) and truncations are indicated by arrows. To analyse different proteins in the same samples simultaneously, membranes were cut accordingly and incubated with respective antibodies separately. For a clear and concise data presentation, displayed blots were cropped slightly.

IRS proteins are crucial mediators of various cellular functions. They exhibit a common architecture comprised of conserved pleckstrin homology (PH) as well as phosphotyrosine binding (PTB) domains and a more variable C-terminus containing tyrosine and serine residues which can be phosphorylated. Depending on the residues phosphorylated and the downstream effectors engaged, specific signalling outcomes are initiated. Apart from general physiological functions such as glucose metabolism, mitogenesis and survival, IRS proteins exert more particular functions in a tissue-specific context^24–26^ (Fig. 1a). The relevance of Insulin Receptor Substrate 4 (IRS4) however has not been clarified yet. Here, we aim to decipher its function as a novel modulator of BMP signalling.

A tandem affinity-based mass spectrometry approach was used to screen for proteins interacting with BMP receptors. We identified several BMP receptor type II (BMPRII) associated proteins, among these IRS4, which co-precipitated with BMPRII long form both in the absence and presence of BMP2 (Supplementary Fig. S1a). BMPRII exists in two splice variants; a short form (BMPRII-SF) ending after the kinase domain and a long form (BMPRII-LF), the prevalent form in most cell types^27^. To confirm the interaction, co-immunoprecipitation experiments using HEK293T cells were performed. HA-tagged BMPRII long form (HA-BMPRII-LF) co-precipitated with endogenous IRS4 as well as with myc-tagged IRS4, whereas in control samples the receptor was not detected (Fig. 1b and c). To analyse whether interaction with BMPRII is specific to IRS4, IRS1–3 were included into the studies. We observed that in addition to IRS4 also IRS2 interacts with BMPRII-LF (Supplementary Fig. S1b), which might be explained by the slightly higher sequence homology of IRS2 with IRS4 compared to the other IRS proteins^1^. Noteworthy, the interaction of IRS4 with BMPRII-LF was independent of BMP2 stimulation (Fig. 1d) indicating that scaffold protein IRS4 is constitutively bound to the receptor. To investigate whether IRS4 interacts with BMPRII at the plasma membrane, cell surface biotinylation assays were performed in transfected HEK293T cells. Indeed, more IRS4-myc was precipitated when HA-BMPRII-LF was co-expressed in these cells (Fig. 1e, compare lanes 1 and 3 upper panel). The faint band appearing when no HA-BMPRII-LF was co-expressed (Fig. 1e, lane 3 upper panel) can be assigned to the binding of IRS4 to other endogenous surface receptors like the insulin, FGF or leptin receptors^28–30^, which were biotinylated as well. In cells expressing IRS1-myc co-expression of BMPRII-LF did not lead to increased signals (Fig. 1e, compare lanes 5 and 7 upper panel) confirming the results from Supplementary Fig. S1b.

IRS4 was identified as a 160 kDa protein undergoing rapid tyrosine phosphorylation upon insulin stimulation^1^. Using a pan-phospho tyrosine antibody, we found strongly increased tyrosine phosphorylation of precipitated IRS4-myc after BMP2 stimulation (Fig. 1f), which provides additional evidence for the role of IRS4 in BMP signalling.

Taken together, IRS4 constitutively associates with BMPRII at the plasma membrane and BMP-mediated receptor activation induces tyrosine phosphorylation of IRS4.

### Mapping of the BMPRII binding site at IRS4

To further characterise the IRS4/BMPRII interaction and to elucidate which region of IRS4 is involved in binding to BMPRII, a series of IRS4 truncations was generated (Fig. 1g). Truncations lacking parts of the C-terminal portion, the PH or PH and PTB domains were characterized for interaction with BMPRII-LF. Serial truncations of IRS4 from the C-terminus to amino acid residue 604 and the IRS4 wild type (WT) revealed clear and robust binding to BMPRII-LF (Fig. 1h). Deletion of the PH domain (ΔPH) or deletion of PH and PTB domain (ΔPH_PTB) abolished the association. Using the shortest truncations containing only the PH domain (E201) or only the PH and PTB domains (Y337), binding to BMPRII was severely affected. Together, these results imply that the PH domain as well as a C-terminal region distal of the PTB domain are required to foster the interaction with BMPRII.

### IRS4 expression causes a decrease in Smad1 protein levels

**Figure 2:**
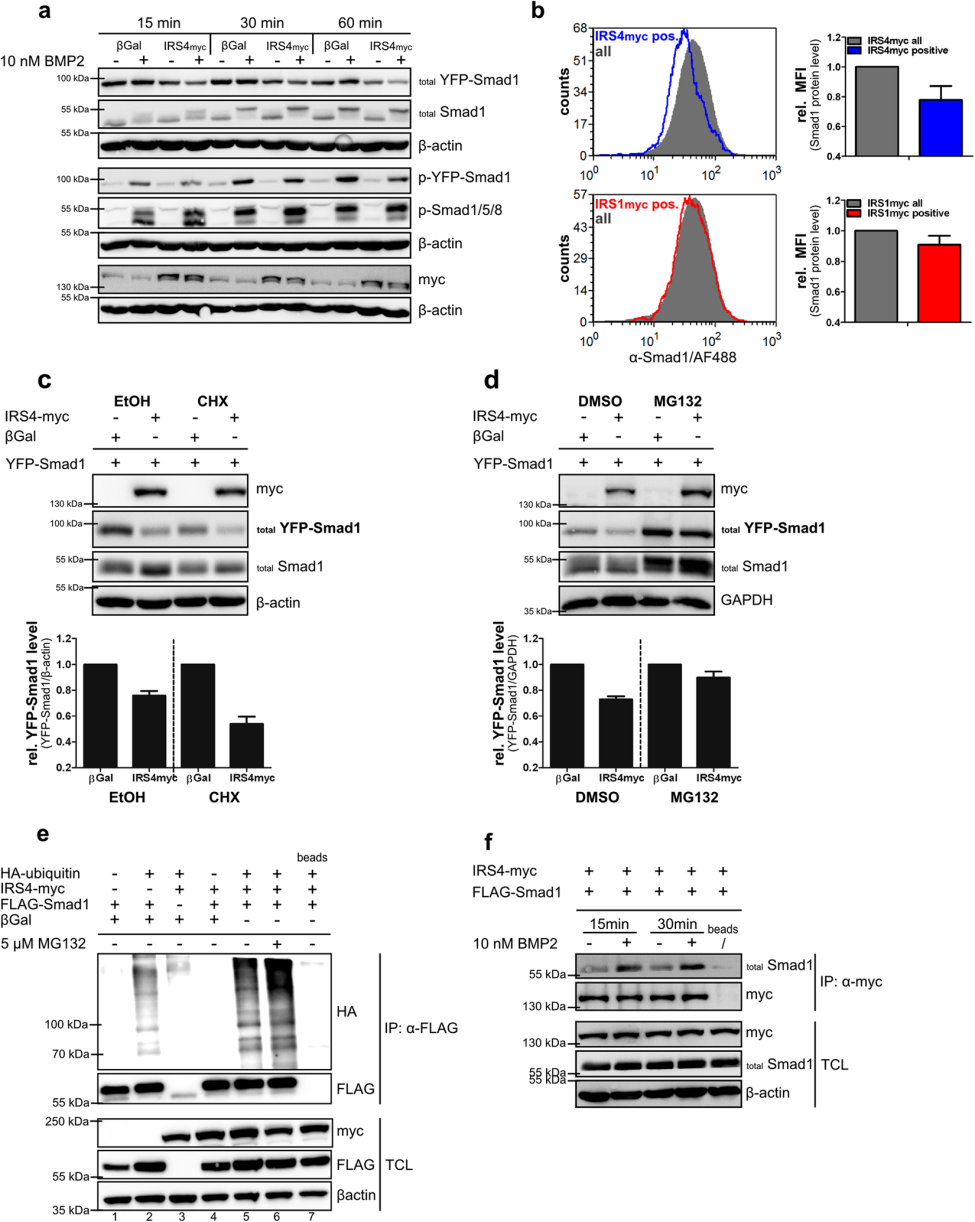
IRS4 causes reduction of Smad1 levels. (**a**) IRS4 expression reduces Smad1 protein. Transfected C2C12 cells were stimulated with 10 nM BMP2 for the indicated times. Lysates were analysed by Western blotting using indicated antibodies. (**b**) IRS4 interferes with endogenous Smad1 protein levels. Transfected C2C12 cells were gated on myc-positive single cells (blue and red curve) via flow cytometry and endogenous Smad1 levels were analysed. Median fluorescence intensity (MFI) values of myc-positive cells were compared to those of all cells (grey curves). Bar charts represent means ± SD of 3 independent experiments. (**c**,**d**) The Smad1 decrease is mediated via proteasomal degradation. Transfected C2C12 cells were incubated with 5 µg/ml cycloheximide (**c**) or 10 µM MG132 (**d**) for 6 h; ethanol and DMSO were applied as respective vehicle control. Lysates were subjected to Western blotting. Quantification depicts total YFP-Smad1 level normalised to β-actin or GAPDH relative to β-galactosidase. Bar charts represent mean ± SD from 3 independent experiments. (**e**) IRS4 enhances ubiquitination of Smad1. Transfected HEK293T cells were incubated with 5 µM MG132 overnight and subjected to immunoprecipitation of FLAG-Smad1 using an α-FLAG antibody. Immunoprecipitates and TCL were analysed by Western blotting using indicated antibodies. Incubation with beads only served as IP control. (**f**) IRS4 interacts with Smad1. Transfected HEK293T cells were stimulated with 10 nM BMP2 for indicated times. Immunoprecipitation was performed using α-myc antibody; precipitates and TCL were analysed by Western blotting. Incubation with beads only served as IP control. To analyse different proteins in the same samples simultaneously, membranes were cut accordingly and incubated with respective antibodies separately. For a clear and concise data presentation, displayed blots were cropped slightly.

To investigate the impact of IRS4 on BMP signalling, we used C2C12 myoblast cells which, in contrast to HEK293T cells, do not express IRS4 endogenously (Supplementary Fig. S2a). To examine the role of IRS4 in BMP-induced phosphorylation of Smads, cells were co-transfected with YFP-Smad1 and IRS4-myc. The molecular weight shift of YFP-fused Smad1 compared to endogenous Smad1/5/8 enables identification of transfected cells, thus allowing a selective analysis. We found that expression of IRS4-myc led to reduced BMP2-induced YFP-Smad1 phosphorylation compared to mock transfected control cells. Concurrently, total YFP-Smad1 protein levels were considerably decreased, which might account for the reduced pYFP-Smad1 levels (Fig. 2a). This finding was substantiated on the level of endogenous Smads using flow cytometry measurements enabling the analysis of transfected cells via specific cell gating. The IRS4-myc-positive gated cell population revealed a marked reduction of endogenous Smad1. This effect was specific to IRS4 as it was not observed for IRS1-myc-positive cells (Fig. 2b). BMP2 stimulation had no impact on the IRS4-mediated Smad1 decrease (Supplementary Fig. S2b). Endogenous Smad5 levels as well as FLAG-Smad5 levels were not affected by IRS4-myc expression (Supplementary Fig. S2c and d) indicating a mechanism specific to Smad1. Overall, IRS4 affects basal Smad1 but not Smad5 protein levels which in turn reduces the Smad1 pool available for ligand-induced phosphorylation.

### The IRS4-dependent decrease of Smad1 protein is due to enhanced ubiquitination and proteasomal degradation

To understand the underlying mechanism of the IRS4-dependent Smad1 reduction in more detail, transfected C2C12 cells were treated with cycloheximide to study Smad1 stability/turnover via inhibition of *de novo* protein synthesis. Cycloheximide treatment caused a more pronounced reduction of YFP-Smad1 protein in IRS4-myc expressing cells when compared to control cells, while FLAG-Smad5 levels remained unaffected (Fig. 2c and Supplementary Fig. S2d). Analogous experiments using MG132 to inhibit proteasomal activity revealed a rescue of the IRS4-induced YFP-Smad1 decrease almost comparable to control cells (Fig. 2d). *In vitro* ubiquitination assays demonstrated enhanced poly-ubiquitination of Smad1 upon IRS4 expression (Fig. 2e, compare lanes 2, 5 and 6). These results strongly suggest that IRS4 supports poly-ubiquitination of Smad1 consequently resulting in its increased proteasomal degradation.

Next, we investigated whether IRS4 interacts with Smad1 and found that FLAG-Smad1 co-immunoprecipitated with IRS4-myc, which was even enhanced by BMP2 stimulation (Fig. 2f). We conclude that IRS4 by binding to Smad1 promotes its ubiquitination thus targeting Smad1 for degradation. The IRS4/BMPRII interaction might facilitate association with Smad1 by ensuring a close proximity of both proteins particularly upon BMP2 stimulation.

### IRS4 reduces the transcriptional activity of Smad proteins

**Figure 3:**
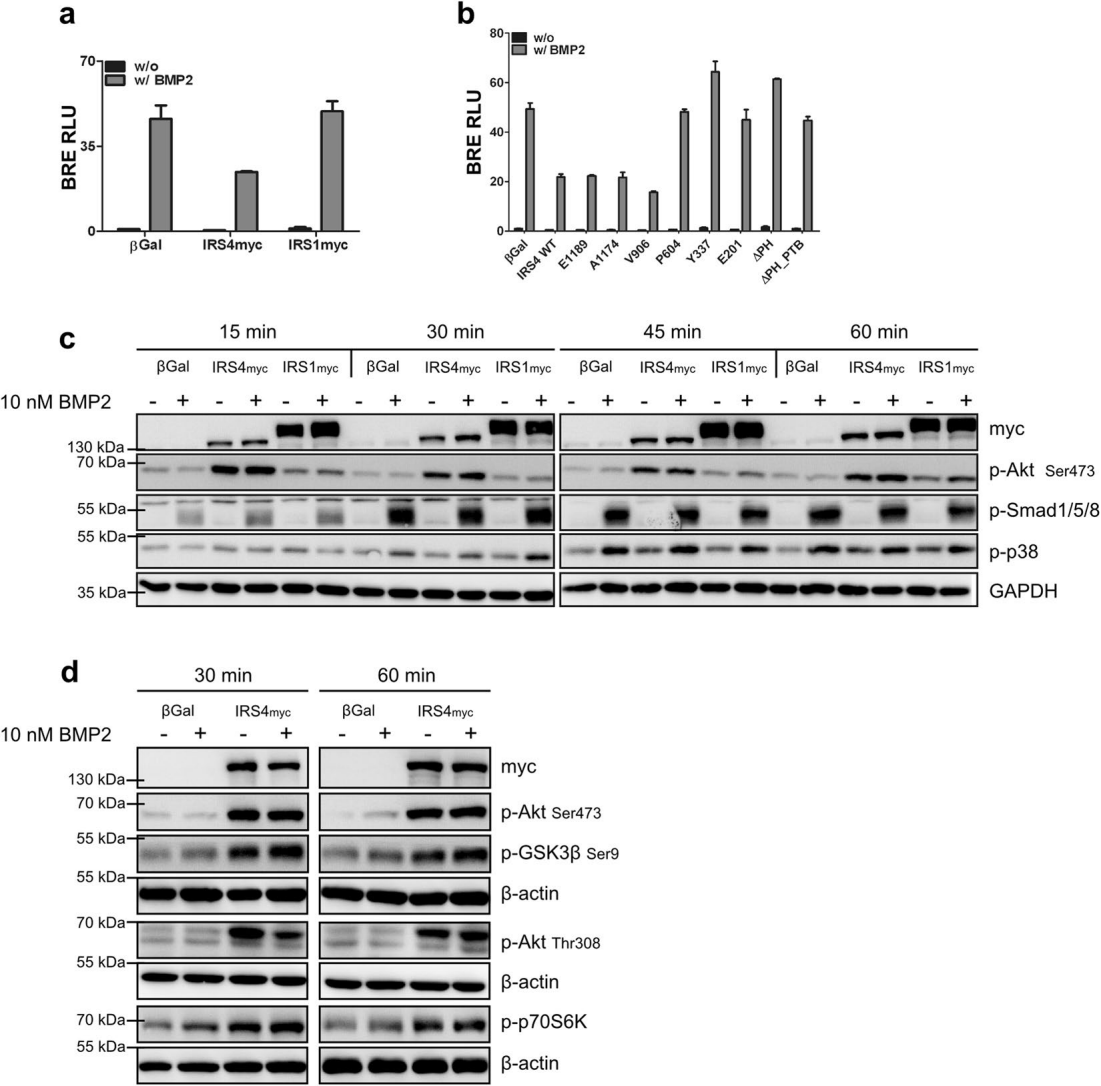
IRS4 reduces transcriptional activity of Smad proteins and initiates Akt pathway activation. (**a**,**b**) IRS4 but not IRS1 affects Smads transcriptional activity. C2C12 cells transfected with BRE-luc, RL-TK and IRS1-myc or IRS4-myc (**a**), truncated mutants thereof (**b**) or β-galactosidase as indicated were stimulated with 5 nM BMP2 for 24 h. Bar charts depict means ± SD of RLU from triplicate measurement relative to β-galactosidase representative for 3 independent experiments. RLU = relative luciferase units (BRE-luc/RLTK-luc). (**c**,**d**) Expression of IRS4 but not IRS1 induces activation of Akt signalling. Transfected C2C12 cells were stimulated with 10 nM BMP2 for indicated times. Lysates were subjected to Western blotting using indicated antibodies. To analyse different proteins in the same samples simultaneously, membranes were cut accordingly and incubated with respective antibodies separately. For a clear and concise data presentation, displayed blots were cropped slightly.

To analyse the functional implication of the IRS4-mediated Smad1 degradation on the expression of BMP/Smad target genes, reporter gene assays were performed using luciferase under the control of a BMP-responsive element (BRE) derived from the murine *Id1* promoter^31^. Expression of IRS4-myc led to significant attenuation of BRE reporter gene activity in response to BMP2 after 6 h (Supplementary Fig. S2e). This effect was even more pronounced after 24 hours of BMP2 stimulation (Fig. 3a). Expression of IRS1-3 did not alter BMP2-induced Smad-dependent transcriptional activity (Fig. 3a and Supplementary Fig. S2f), again underlining the IRS4-specific effect. Moreover, IRS4-myc expression resulted in decreased ID1 protein levels compared to control cells (Supplementary Fig. S2g). Taken together, the observed reduction of Smad1 protein in the presence of IRS4 diminishes BMP/Smad-mediated transcription.

To characterise the mechanism of interference in more detail and to gain insights into the domains of IRS4 responsible for these effects, the aforementioned IRS4 truncations were included in functional studies (Fig. 1g). Myc-tagged E1189- and A1174 truncations inhibited BRE reporter gene activity comparable to IRS4-myc wild type, while the V906 truncation showed a slightly stronger inhibition after BMP2 stimulation. In contrast, the other truncations tested did not affect BMP2-induced Smad-dependent transcriptional outcome (Fig. 3b and Supplementary Fig. S2h).

Our data suggest that it is the C-terminal portion of IRS4, which is important for the protein’s impact on BMP signalling. In addition, the PH domain turned out to be crucial for the IRS4-mediated inhibition of BMP/Smad signalling which is in accordance with our interaction studies indicating its requirement for the association with BMPRII.

### Expression of IRS4 is linked to strong Akt activation

Next, we studied the impact of IRS4 on BMP/non-Smad signalling. While expression of IRS4-myc and IRS1-myc did not affect BMP2-induced phosphorylation of MAPK p38, there was a strong and ligand-independent phosphorylation of Akt at residue Ser473 in the presence of IRS4-myc only (Fig. 3c). The effect of IRS4 on Akt activation has been described previously for various cancer cell lines, fibroblasts, myeloid progenitor cells, HepG2 and HEK293T cells^6, 7, 32–35^. Here, we demonstrate the link between IRS4 and Akt signalling in muscle precursor cells. Expression of IRS4-myc in C2C12 cells induced phosphorylation of Akt at residue Thr308 and Ser473, critical for full activation of Akt^36^ (Fig. 3d). Downstream targets of Akt such as p70S6K and GSK3β were phosphorylated as a consequence of IRS4-myc expression. We conclude, that the IRS4-dependent decrease of Smads transcriptional activity along with a strong activation of the Akt pathway might have an impact on the differentiation of myogenic precursor cells.

### IRS4 is expressed in developing mouse limbs, primary foetal mouse myoblasts and postnatal satellite cells

**Figure 4:**
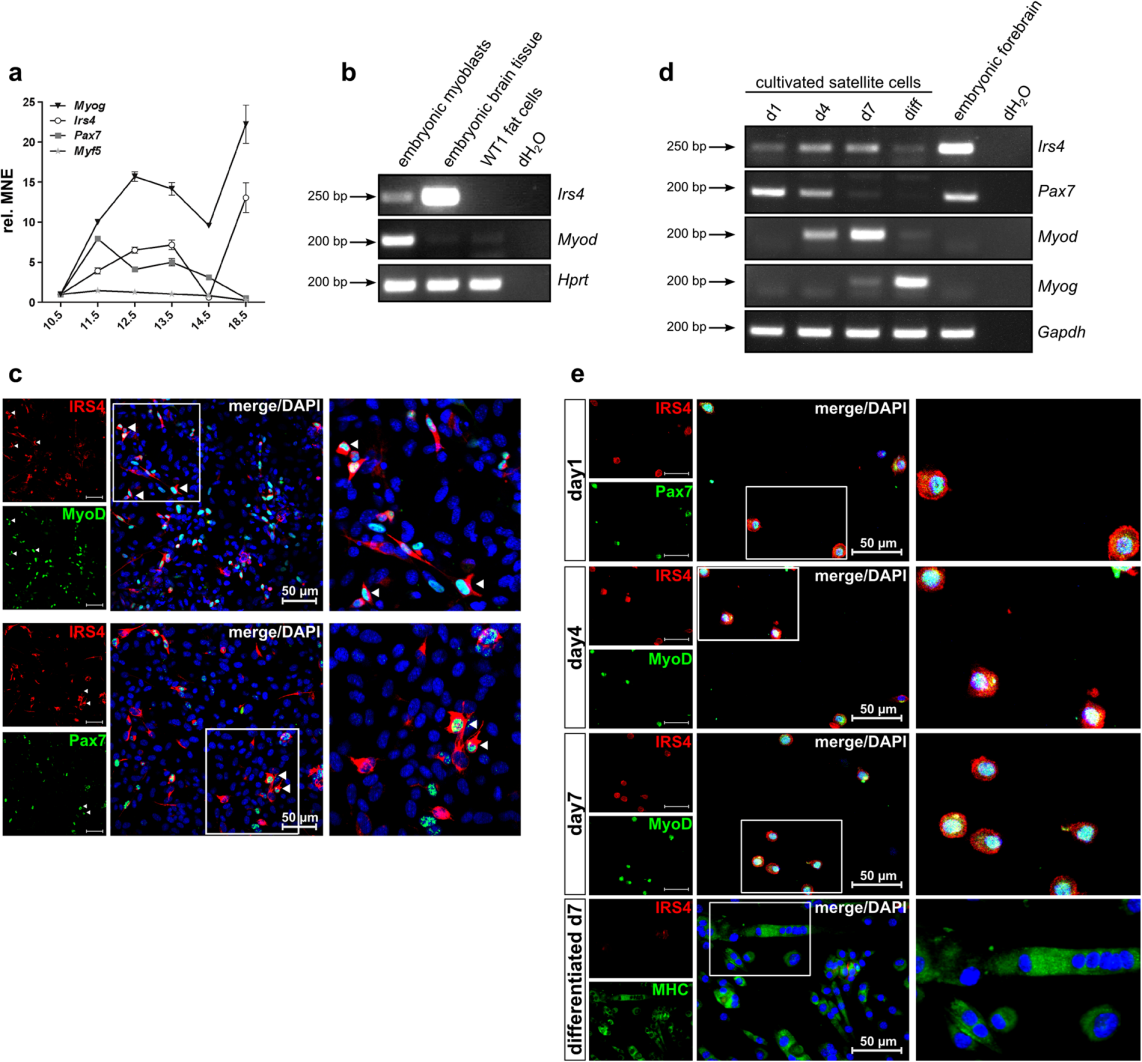
*In vivo* expression of IRS4 in mouse limbs, primary myoblasts and satellite cells. (**a**) IRS4 is expressed during mouse limb development. RNA from mouse limbs of indicated embryonic developmental stages was isolated, reverse-transcribed and subjected to gene expression analyses via qRT-PCR. Data points summarise triplicate measurements and depict mean normalised expression (MNE) ± SEM representative for 2 independent experiments. (**b**) IRS4 is expressed in cells freshly isolated from foetal limb skeletal muscle. Primary mouse myoblasts were isolated from E18.5 limb muscles. PCR shows *IRS4* transcript in myoblasts; E14.5 embryonic brain tissue served as positive control, WT1 fat cells as negative control. (**c**) Immunofluorescence staining of primary E18.5 mouse myoblasts using indicated specific antibodies shows IRS4/MyoD and IRS4/Pax7positive cells (see arrows); nuclei were stained using DAPI. (**d**) IRS4 is expressed in cultured satellite cells. Mouse satellite cells were isolated from P7 limb muscles, cultured for the indicated days and subjected to PCR analyses. Embryonic forebrain served as positive control. (**e**) Immunofluorescence staining of cultured mouse satellite cells using indicated specific antibodies reveals expression of IRS4 in postnatal muscle precursor cells; nuclei were stained using DAPI.

To confirm our data *in vivo*, we next analysed the expression of *IRS4* in developing mouse limbs. Using whole limb mRNA, *Irs4* expression was detectable in limb mesenchyme and was upregulated during embryonic myogenesis between stage E10.5 and E13.5, whereas at E14.5 mRNA levels were strongly decreased. IRS4 showed robust expression again in foetal limb tissue at E18.5 (Fig. 4a and Supplementary Fig. S3a). While the expression of the early myogenic markers Pax7 and Myf5 decreased at later developmental stages (Fig. 4a and Supplementary Fig. S3b and c), *MyoG* levels increased over time. Remarkably, *Myog* expression revealed a similar trend as *IRS4* mRNA levels (Fig. 4a and Supplementary Fig. S3a and d). Next, we analysed which limb mesenchymal cells express IRS4 *in situ* via immunostaining on tissue sections, however of several tested antibodies none provided reliable results. We therefore isolated primary myoblasts from E18.5 developing mouse limb muscles which showed clear expression of *Irs4* mRNA (Fig. 4b). For immunolabelling, cells were immediately cytospun and fixed to reflect the endogenous *in vivo* situation as closely as possible and to avoid culturing artefacts. After cytospin, cells were subjected to immunofluorescence staining for IRS4, MyoD and Pax7. As expected the cells constituted a mixed population with myoblasts positive for MyoD and/or Pax7. Cells positive for nuclear MyoD or Pax7 staining revealed a co-expression of cytosolic IRS4 protein (Fig. 4c, see arrows). These findings were further confirmed in mouse satellite cells isolated at postnatal day 7. Cultivation of these cells induced commitment into myoblasts characterised by upregulation of *Myod* mRNA as early differentiation marker and decrease of the satellite cell marker *Pax7*. Noteworthy, *Irs4* levels were also increased during cultivation correlating with *Myod* expression. In myoblasts at the brink of terminal differentiation, i.e. high in *Myod* and initiating *Myogenin* expression, *Irs4* expression peaked. After that IRS4 expression decreased again in terminally differentiated myoblasts/myotubes exclusively positive for Myogenin (Fig. 4d). In line, immunofluorescence stainings showed co-expression of IRS4 with both Pax7 and MyoD in cultured satellite cells. In contrast, IRS4 protein was not detected in myotubes (Fig. 4e). Our data demonstrate for the first time co-expression of IRS4 with skeletal muscle markers in intact, freshly isolated myogenic cells.

### IRS4 enhances the expression of myogenic markers thereby triggering myogenesis

**Figure 5:**
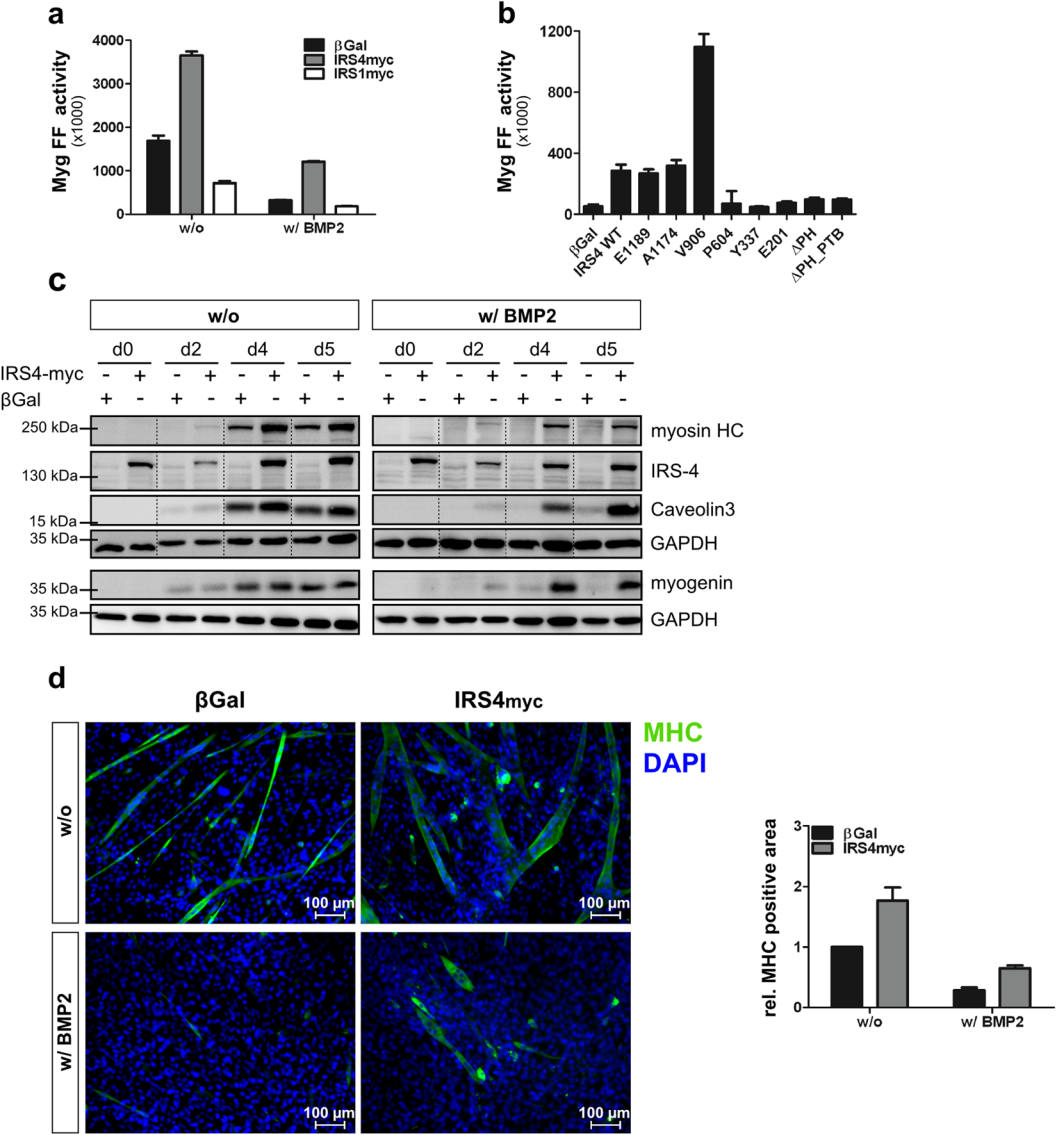
IRS4 enhances expression of myogenic markers and promotes myogenesis. (**a**,**b**) IRS4 but not IRS1 affects Myg-luc expression. C2C12 cells transfected with the Myg-luc reporter and indicated constructs were differentiated with or without 5 nM BMP2. Bar charts depict means ± SD of firefly luciferase activity from triplicate measurements representative for 3 independent experiments. (**c**) IRS4 enhances expression of myogenic markers. Transfected C2C12 cells were differentiated with or without 5 nM BMP2 for indicated times. Lysates were subjected to Western blotting using indicated antibodies. Dashed lines indicate the exclusion of irrelevant lanes from the same membrane (**d**) IRS4 triggers myogenesis. Transfected C2C12 cells were differentiated with or without 5 nM BMP2. Myosin heavy chain (MHC) was stained using a specific antibody; nuclei were stained using DAPI. Images were quantified using ImageJ. Bar chart depicts means ± SD of at least 60 images per condition derived from 4 independent experiments. To analyse different proteins in the same samples simultaneously, membranes were cut accordingly and incubated with respective antibodies separately. For a clear and concise data presentation, displayed blots were cropped slightly.

Based on the endogenous expression of IRS4 in mouse muscle precursor cells and taking the prominent role of Akt and BMP signalling in regulating myogenesis into account, we next investigated the effect of IRS4 in myoblast differentiation. We performed reporter gene assays using luciferase under the control of the myogenin promoter (Myg-luc)^37^. Myogenin is pivotal for terminal differentiation of committed myoblasts into contractile myotubes^38^. Expression of IRS4, but not IRS1, strongly increased the Myg-luc reporter activity (Fig. 5a). While BMP2 treatment during differentiation considerably inhibited reporter activity in control cells, IRS4 transfected cells were still capable of inducing Myg-luc transcription. Using the IRS4 truncations, we found that the E1189 and A1174 truncations promoted Myg-luc activity similar to wild type IRS4, while the V906 variant of IRS4 showed an increased activity with an even more pronounced positive effect on Myg-luc expression as compared to wild type (Fig. 5b). The other truncations showed no impact on Myg-luc reporter activity. These data are in line with the findings derived from the BRE-luc studies (Fig. 3b), i.e. those IRS4 variants that revealed an inhibitory effect on BMP/Smad signalling were demonstrated to promote myogenic differentiation.

To further substantiate our evidence that IRS4 counteracts BMP action and is involved in myogenic differentiation, the expression of the myogenic marker proteins caveolin-3, MyoG and myosin heavy chain (MHC) was analysed. Myogenic markers were stronger expressed in IRS4-myc expressing compared to control cells during the course of differentiation already after two days (Fig. 5c; left panels). While BMP2 stimulation almost completely abolished expression of myogenic markers in control cells, IRS4-myc wild type expressing cells still displayed remarkable expression of analysed marker proteins (Fig. 5c; right panels). The pro-myogenic effect of IRS4 could also be demonstrated by immunofluorescence staining of MHC in C2C12 cells after three days of differentiation. IRS4-myc expression promoted the formation of longer and thicker, i.e. hypertrophic myotubes in the absence of exogenous BMP stimulation. In BMP2-stimulated samples IRS4 was capable of rescuing myogenic differentiation to some extent (Fig. 5d). The enhanced differentiation as indicated by MHC-positive myotubes was confirmed by quantification of the overall MHC-positive area (Fig. 5d, bar chart).

### Targeted knockdown of IRS4 in human myoblasts leads to enhanced BMP signalling and impaired myogenic differentiation capacity

**Figure 6:**
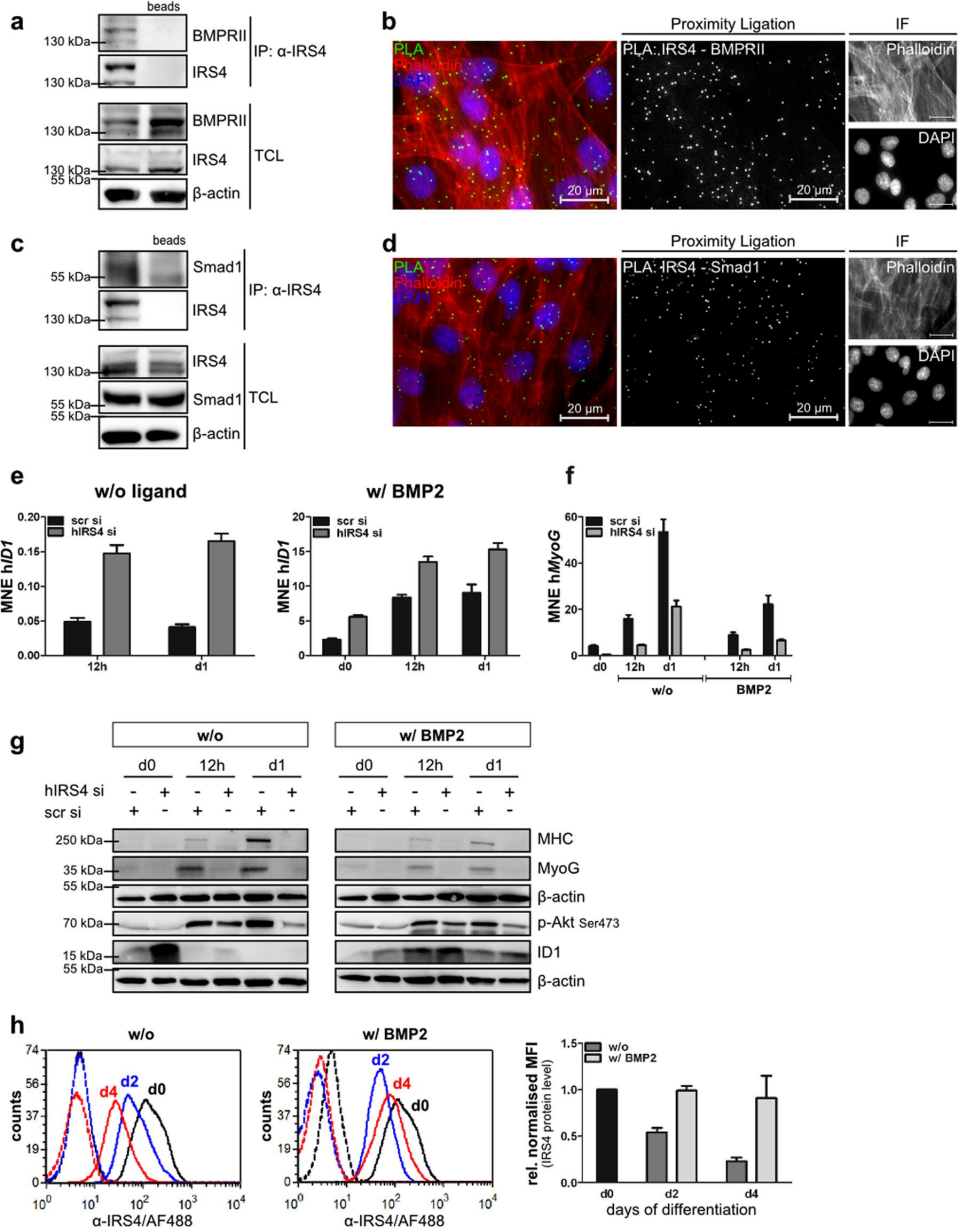
Targeted IRS4 knockdown causes impaired myogenic differentiation capacity in human myoblasts. (**a**,**c**) Endogenous interaction of IRS4 with BMPRII and Smad1. Human myoblasts were subjected to immunoprecipitation using an α-IRS4 antibody. Immunoprecipitates (IP) and total cell lysate (TCL) were analysed by Western blotting using indicated antibodies. Incubation with beads only served as IP control. (**b**,**d**) *In situ* proximity ligation assay (PLA) of IRS4 and BMPRII or Smad1. Human myoblasts were subjected to *in situ* PLA (green signal) to visualize the endogenous association of IRS4 with BMPRII (**b**) or Smad1 (**d**); nuclei and the actin cytoskeleton were stained using DAPI and Phalloidin594. Relevant controls are depicted in Supplementary Fig. S4g. (**e–g**) IRS4 knockdown affects myogenesis and BMP signalling. Human myoblasts were transfected with siRNA targeting either nonspecific sequences (scr si) or human IRS4 (hIRS4 si) and differentiated with or without 10 nM BMP2 for indicated times. Gene expression was analysed by qRT-PCR. Bar charts summarise triplicate measurements and depict MNE ± SEM representative for 3 independent experiments. (**g**) Protein lysates were subjected to Western blotting using indicated antibodies. (**h**) IRS4 expression decreases during differentiation. Human myoblasts were differentiated with or without 10 nM BMP2 for indicated times. IRS4 expression was assessed by flow cytometry using a specific antibody (solid line) or isotype control IgG (dotted line). Bar charts depict means ± SD of MFI values normalised to appropriate IgG control relative to d0 of 3 (w/o) or 2 (w/) independent experiments, respectively. To analyse different proteins in the same samples simultaneously, membranes were cut accordingly and incubated with respective antibodies separately. For a clear and concise data presentation, displayed blots were cropped slightly.

To complement our results obtained from gain-of-function experiments in IRS4 overexpressing C2C12 cells, we performed loss-of-function experiments in immortalised human myoblasts^39^. Initially, these cells were characterised with regard to their BMP signalling properties. As depicted in Supplementary Fig. S4a, immortalised human myoblasts express BMP type I and type II receptors. Stimulation with BMP2 induced Smad1/5/8 phosphorylation and induction of *ID1* mRNA, but also phosphorylation of MAPK p38 (Supplementary Fig. S4b and c). BMP2 treatment efficiently prevented these cells from differentiating into myotubes (Supplementary Fig. S4d). Next, endogenous expression of IRS4 was verified via flow cytometry measurements (Supplementary Fig. 4e). Using co-immunoprecipitation analyses, we demonstrated endogenous interaction of IRS4 with BMPRII and Smad1 (Fig. 6a and c). This was validated by an *in situ* proximity ligation assay (PLA) clearly visualising the IRS4/BMPRII and IRS4/Smad1 association in intact myoblasts (Fig. 6b and d). To further examine the biological relevance of IRS4 in both BMP signalling and in myogenic differentiation, IRS4 was depleted by siRNA-mediated knockdown. Verification of knockdown efficiency clearly demonstrated depletion of both IRS4 mRNA and protein (Supplementary Fig. 4f). Knockdown of IRS4 revealed a marked increase of ID1 mRNA and protein during the course of myogenic differentiation particularly at day 0. BMP2 stimulation induced ID1 expression whereas IRS4 depleted cells showed an even stronger induction (Fig. 6e and g). MyoG and MHC, which were upregulated during myogenesis and repressed by BMP2 addition, were only barely detectable when IRS4 was depleted (Fig. 6f and g). Comparably, IRS4 knockdown in either primary mouse foetal myoblasts or postnatal satellite cells caused decreased myotube formation as assessed by MHC and actin staining (Supplementary Fig. S5a and b). Furthermore, depletion of IRS4 led to reduced phosphorylation of Akt at any time point analysed independent of ligand addition, while control cells displayed a higher pAkt level especially after induction of differentiation. Interestingly, we found that IRS4 protein was successively downregulated during myogenesis. In contrast, protein levels remained almost unchanged when myogenic differentiation was prevented by BMP2 treatment (Fig. 6h). This is in accordance with our findings in satellite cells in which IRS4 was expressed during proliferation but not in differentiated myotubes (Fig. 4d and e).

Taken together, our data demonstrate that IRS4 is a pivotal factor in myogenesis. IRS4 is expressed in myoblasts during mouse limb development and its expression level strongly correlates with the cells’ state of differentiation. Furthermore, IRS4 depletion resulted in impaired myogenic differentiation capacity. We propose that this is due to IRS4-mediated downregulation of BMP/Smad concomitant to enhanced PI3K/Akt signalling.

## Discussion

BMP ligands elicit critical biological outcomes by initiating both the canonical Smad pathway as well as non-Smad mediated routes strongly depending on the cellular context. Considering the functional diversity of BMPs, BMP signalling is subject to tight regulation ensuring a concise and balanced propagation of signalling responses. Hence, a multitude of elaborate regulatory mechanisms facilitate physiological BMP functions. In the last years, various BMP receptor interacting proteins have been reported to affect BMP signal transduction by distinct modes of action^19, 20, 40–42^. Still, it is of great importance to identify modulatory proteins to explain specific cell-context dependent actions of this growth factor family. Analysing regulatory networks provide the knowledge of subtle fine-tuning, signalling crosstalk and specificity, which is of crucial relevance particularly for understanding complex processes such as tissue regeneration.

**Figure 7:**
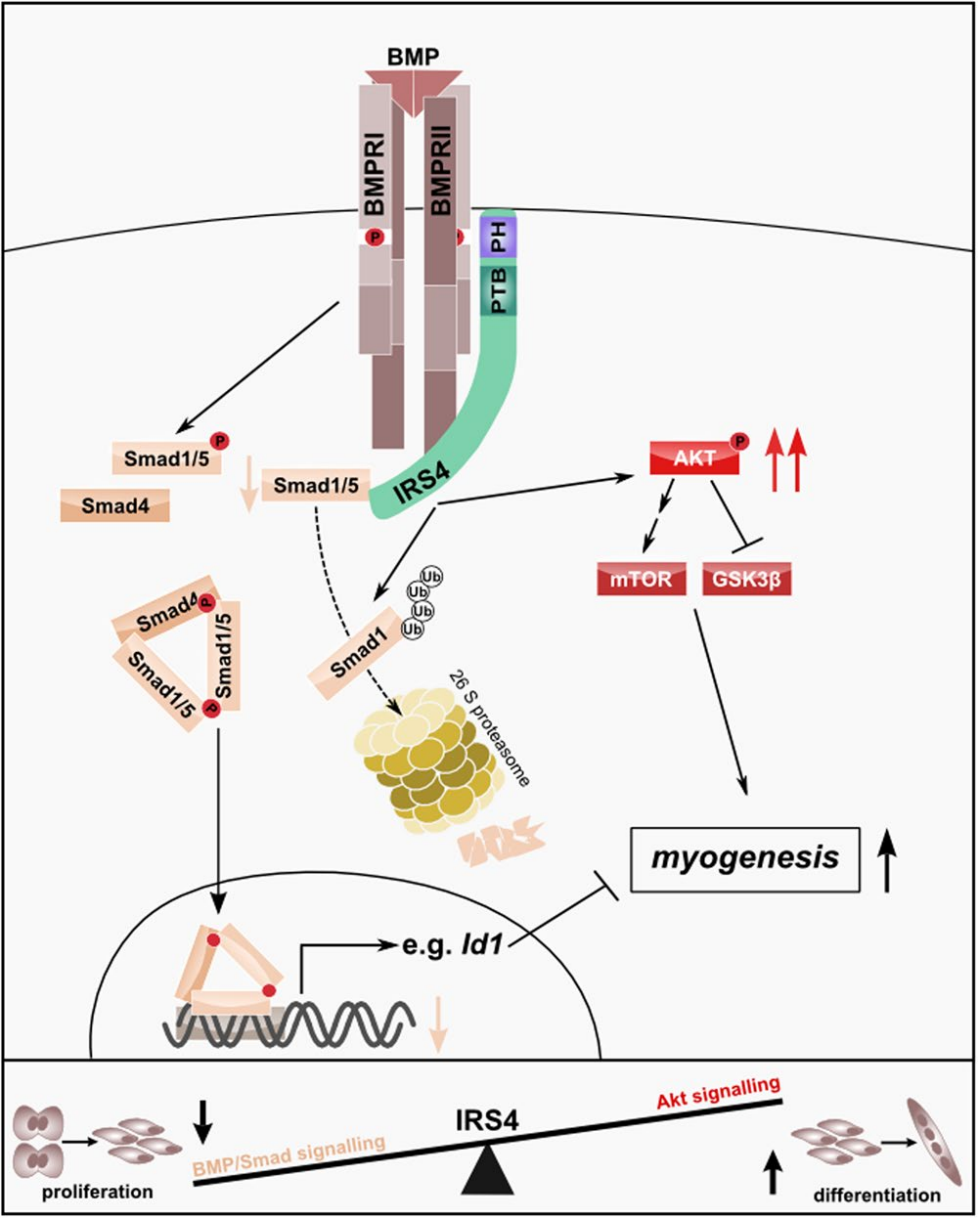
IRS4 interferes with BMP signal transduction and functions as a novel regulator of BMP signalling and myogenesis. IRS4 interacts with BMPRII in a ligand independent manner and affects BMP signalling outcomes. Presence of IRS4 in cells results in reduced Smad1 protein levels, while Smad5 levels are unaffected. This is due to enhanced poly-ubiquitination and subsequent proteasomal degradation of Smad1, which consequently results in reduced transcriptional activity of BMP Smads. In addition, IRS4 provokes activation of the PI3K/Akt signalling axis. Both, the IRS4-dependent inhibition of BMP/Smad signalling and the IRS4-mediated Akt pathway activation eventually contribute to the differentiation of muscle precursor cells into the myogenic lineage.

Here, we present Insulin Receptor Substrate 4 (IRS4) as a novel regulatory factor in the BMP pathway and provide a new molecular mechanism of this scaffold protein. We show that IRS4 interferes with BMP signalling by interacting with the BMP receptor BMPRII and by promoting ubiquitination and proteasomal degradation of specifically Smad1. Concomitantly, IRS4 activated the PI3K/Akt axis which in concert with decreased BMP/Smad signalling enhanced myogenic differentiation of muscle precursor cells (illustrated in Fig. 7).

Within a proteomic screen for novel proteins interacting with BMPRII, we identified the adapter protein IRS4. We used different immunoprecipitation-based approaches and *in situ* PLA to demonstrate that the interaction of both proteins was very robust and occurred at the plasma membrane. Noteworthy, the interaction was independent of BMP2 stimulation. In contrast, other BMPRII interacting proteins like cGKI and Tribbles-like protein 3 (Trb3) are reported to dissociate from the receptor upon ligand addition either to enhance transcriptional responses by binding to activated Smads or to trigger degradation of Smurf1 thus potentiating the BMP/Smad pathway^20, 43^. Interaction of the rare PI3K regulatory subunit p55γ with BMPRII is enhanced by BMP2 leading to ligand-induced chemotaxis of progenitor cells^41^. IRS4 as a classical scaffold protein does not exhibit any intrinsic catalytic activity but rather serves as binding hub between cell surface receptors and downstream signalling components. We propose that even though binding to BMPRII is not altered by BMP2, ligand addition might modify the overall platform of binding sites within IRS4. Upon stimulation with insulin and leptin, IRS4 was reported to be tyrosine phosphorylated thereby giving rise to binding of e.g. SH2 domain containing proteins like PI3K and Grb2^2, 29^. Interestingly, we found that IRS4 was also phosphorylated on tyrosine residues when cells were stimulated with BMP2. This might provoke engagement of a different subset of downstream proteins hence influencing signalling outcome. Furthermore, this finding might point to the dual kinase activity of BMPRII, which has already been proposed^41, 44^ and was described for the related TGFβ receptor type I and II^45, 46^. It is tempting to speculate that other BMP receptor-associated kinases such as c-kit or c-src mediate BMP2-induced tyrosine phosphorylation of IRS4^47–49^.

Mapping of the BMPRII binding site at IRS4 revealed the PH domain and a C-terminal region distal of the PTB domain to mediate the association. We assume that the PH domain is required to recruit the protein to the plasma membrane by binding to phospholipids thereby enabling close proximity to BMPRII, while the association ﻿with the receptor is mediated via the C-terminus. Beyond, consideration might be given that the PH domain is capable of directly binding to upstream receptors as suggested by a study mapping the IRS4 interaction with the leptin receptor^29^. IRS proteins bind to pTyr within receptors via their PTB domain^25^. Recently, the BMPRII was shown to be tyrosine phosphorylated^41^ thus providing a pivotal feature for interacting with IRS4. Still, based on our data, we cannot assure that the PTB domain is needed for the association or comment on whether the interaction is direct or mediated via any intermediate proteins linking IRS4 to BMPRII.

To investigate the functional implication of the IRS4/BMPRII interaction on BMP signalling, kinetic analyses and reporter gene assays were performed. We found that expression of specifically IRS4 caused decreased total Smad1 protein levels in C2C12 cells. We provide clear evidence for a mechanism involving IRS4-driven enhanced poly-ubiquitination and proteasomal degradation which appeared to be Smad1-specific since Smad5 levels were not affected. These findings suggest that Smad1 and Smad5 display divergent roles in propagating BMP signalling in C2C12 cells, as Smad5 was not sufficient to compensate for the loss of Smad1. BMP Smads are highly homologous and exert overlapping functions referring to different studies^50–55^. However, evidence emerges that Smads do not exclusively function redundantly and regulate distinct cellular functions depending on the cellular context^56–58^. Various modes of interference with R-Smad activity such as C-terminal dephosphorylation catalysed by distinct phosphatases^40, 59^, sequential phosphorylation in the Smad linker region by MAPK/CDKs and GSK3β triggering poly-ubiquitination and degradation^60^ but also inhibitory Smads6/7 targeting R-Smads for degradation in a negative feedback manner^61, 62^ are well described. Here, we demonstrate a novel regulatory mechanisms leading to IRS4-mediated reduced Smad1 stability and thus decreased overall Smad1 pool in the absence of ligand. We speculate that scaffold protein IRS4 does not only interact with BMPRII and Smad1 but also associates with and thereby recruits components intimately linked to the ubiquitination/proteasomal machinery. In conclusion, IRS4 expression fundamentally reduces steady-state Smad1 protein resulting in decreased transcriptional activity of Smads and thus repressed BMP/Smad signalling outcome.

In recent years, studies on the role of BMPs in muscle maintenance and regeneration emerged remarkably. For example, it has been described that BMP signalling promotes proliferation of muscle precursor cells, while differentiation is rather inhibited^14, 63–66^. This blockage of differentiation is mediated by BMP-induced ID proteins, which bind to E-proteins thereby preventing the formation of transcriptional active heterodimers with myogenic regulatory factors^67, 68^. Furthermore, the BMP/Smad axis has been shown to exert positive effects on the regulation of muscle mass thus emphasizing a critical role for BMP signalling in muscle homeostasis^69, 70^. We found that expression of IRS4, by counteracting BMP/Smad signalling, efficiently triggered myogenic differentiation of C2C12 cells. Concurrently, IRS4 knockdown in human myoblasts revealed an upregulation of ID1, while myogenic differentiation capacity was severely impaired. We also confirmed this in primary muscle precursor cells by depletion of IRS4. Of note, the pro-myogenic impact of IRS4 even appeared in the absence of exogenously added BMP, while the medium added for differentiation might contain some BMPs. Our data strongly suggest that the promoting effect of IRS4 on myogenic differentiation might be a result of both inhibition of BMP/Smad signalling and at the same time activation of the Akt pathway. Akt is a critical player during myogenic differentiation and triggers protein synthesis by activating the mTOR/p70S6K axis which is pivotal for not only muscle differentiation but also hypertrophy^71^. Simultaneously, active Akt inhibits GSK3β which itself is a potent suppressor of myogenesis^72, 73^. In line, we demonstrated that knockdown of IRS4 resulted in decreased Akt activation and lack of myotube formation. Noteworthy, we found IRS4 protein levels in human myoblasts to be intimately linked to the cells’ differentiation state: Once cells were terminally committed to the myogenic differentiation programme, as indicated by robust expression of Myogenin, IRS4 protein was downregulated while IRS4 level remained unaltered upon BMP2 treatment, indicating a critical role for IRS4 in regulating myogenic progenitor differentiation. Comparable results were observed in cultured and differentiated primary satellite cells. In conclusion, the pro-myogenic effect of IRS4 is likely due to a combination of BMP/Smad inhibition decreasing ID1 expression and alleviating its inhibitory effect on myogenic regulatory factors, and in addition interference of IRS4 with other pathways pivotal for myogenesis, e.g. switching BMP signalling to the Akt pathway. Our study demonstrates the important role of IRS4 in differentiation of muscle precursors for the first time and strongly emphasizes the relevance of IRS4 in skeletal muscle, one of the few tissues where it has been detected^4, 9^. Male IRS4 knockout mice exhibited significantly reduced weight pointing to a muscle phenotype and indicates a role for IRS4 as a regulator of muscle differentiation/homeostasis^11^. This is further supported by our *in vivo* expression data of IRS4 in mouse myoblasts during embryonic and foetal development as well as in mouse satellite cells.

Taken together, our results give the first functional evidence for IRS4 as a critical regulator of BMP/Smad and Akt signalling in the context of muscle differentiation. This represents a new mechanism explaining cell-context specific actions of BMPs as IRS4 serves as a molecular switch in early myogenic differentiation and provides implications in muscle regeneration and maintenance.

## Methods

All methods were carried out in accordance with relevant guidelines and regulations. All experimental protocols were approved by named institutional and/or licensing committees.

### Expression plasmids

The coding sequences of mouse IRS1/2/4 and rat IRS3 were subcloned from Addgene constructs (plasmid# 11374/11373/11363/11361) into the pcDNA3.1 V5/His-TOPO vector. IRS4 truncations were generated using the TOPO TA cloning kit (Invitrogen) according to manufacturer’s instructions. Used primers are listed in Supplementary Table S1. All constructs contain a C-terminal myc-tag.

Plasmids encoding human HA-BMPRII-LF and human Smad1 subcloned into pEYFP-C1 were described previously^74–76^. Human FLAG-tagged Smad1 and murine FLAG-tagged Smad5 were kindly provided by Peter ten Dijke, Leiden, Netherlands. β-galactosidase (βGal) was obtained from Harvey F. Lodish, Cambridge, MA. HA-ubiquitin was kindly provided by Aristidis Moustakas, Uppsala, Sweden.

### Cell culture and transfection

C2C12 mouse myoblasts and HEK293T cells were cultivated in Dulbecco’s modified Eagle Medium (DMEM; Biochrom AG) supplemented with 10% fetal calf serum (FCS; Biochrom AG), 2 mM L-glutamine and penicillin (100 units/ml)/streptomycin (10 µg/ml) (PAA) at 37°C and 10% or 5% CO_2_, respectively. Immortalised human myoblasts were cultured in skeletal muscle growth medium (Provitro) supplemented with supplement mix (Provitro), 50 ng/ml amphotericin, 50 µg/ml gentamicin, 10% FCS, 2 mM L-glutamine and penicillin (100 units/ml)/streptomycin (10 µg/ml) at 37°C and 5% CO_2_.

For transient transfection of HEK293T cells polyethylenimine (PEI; Sigma-Aldrich) was used. C2C12 cells were transfected using Lipofectamine2000 (Invitrogen) according to manufacturer’s instructions. For siRNA-mediated knockdown of human IRS4 Lipofectamine RNAiMAX (Invitrogen) was used according to manufacturer’s instructions; as control non-targeting siRNA was used (for detailed information see Supplementary Methods). In short, cells were transfected with 25 nM siRNA on two consecutive days and experiments were performed 72 h post-transfection.

### Mice and isolation of primary myoblasts and satellite cells

C57BL/6 mice were kept in the animal facility of the Max Planck Institute for Molecular Genetics, Berlin, Germany, in accordance with legal guidelines. All animal procedures conducted within this study were approved by the responsible authority (Landesamt für Gesundheit und Soziales Berlin, LaGeSo) under license number ZH120. Timed matings were set up, and mice were sacrificed by cervical dislocation at indicated days of pregnancy, foetuses were sacrificed by decapitation. Limbs were removed and subjected to RNA extraction and subsequent quantitative real-time PCR analysis or isolation of primary myoblasts as described in Supplementary Methods. For the isolation of primary satellite cells, forelimb and hindlimb muscles from postnatal mice (day 7; P7) were used. Cell isolation, cultivation and differentiation are described in detail in Supplementary Methods.

### Western blotting

Protein lysates were separated by SDS-PAGE and subsequently transferred on nitrocellulose membranes by Western blotting. Membranes were incubated with indicated primary antibodies overnight at 4°C according to manufacturer’s instructions. Antibodies are listed in Supplementary Methods. Chemiluminescent reactions were processed using Femto-Glo ECL reagents (PJK) and documented on a ChemiSmart5000 digital imaging system (Vilber-Lourmat). Proper loading controls (GAPDH or β-actin as indicated) were examined on the same membrane. Images were quantified using Bio1D software (Vilber-Lourmat).

### Co-immunoprecipitation and tyrosine phosphorylation analyses

24 h post-transfection HEK293T cells were either lysed directly or starved for 6 h in serum-free DMEM and stimulated with 10 nM BMP2 or 100 nM insulin (Roche). Immortalised human myoblasts were lysed 48 h post-seeding. Cells were lysed in RIPA buffer (150 mM NaCl, 50 mM Tris/HCl pH 7.4, 0.1% SDS, 0.5–1% NP-40) or TNE buffer 1 (150 mM NaCl, 20 mM Tris pH 7.4, 2 mM EDTA, 1% Triton X-100) supplemented with protease/phosphatase inhibitors (1 mM PMSF, 2 mM Na_3_VO_4_, 20 mM Na_4_P_2_O_7_, 50 mM NaF, complete protease inhibitor cocktail (Roche)). Immunoprecipitation was performed with 1–4 µg anti-myc (9E10, homemade), anti-IRS4 (EP907Y) or anti-IRS4 (Santa Cruz) antibody overnight followed by incubation with protein A coupled sepharose beads (GE Healthcare). Samples were washed with lysis buffer, eluted with Laemmli buffer and subjected to Western blotting.

### Surface biotinylation assay

24 h post-transfection HEK293T cells were incubated with 0.5 mg/ml EZ-Link^™^ Sulfo-NHS-SS-Biotin solution (Thermo Scientific) followed by incubation with 50 mM Tris pH 8.0. Cells were lysed in RIPA buffer2 (150 mM NaCl, 25 mM Tris/HCl pH 7.4, 0.1% SDS, 0.5% NP-40) and pull-down of biotinylated surface proteins was performed overnight using streptavidin coupled sepharose beads (GE Healthcare). Samples were washed with lysis buffer, eluted with Laemmli buffer and subjected to Western blotting.

### *In vitro* ubiquitination assay

24 h post-transfection HEK293T cells were incubated with 5 µM MG132 (Sigma-Aldrich) overnight. Then, cells were lysed in TNE buffer 2 (150 mM NaCl, 20 mM Tris pH 7.4, 10 mM EDTA, 0.5% Triton X-100, 0.5% sodium deoxycholate) supplemented with complete protease inhibitor cocktail and 10 mM N-ethylmaleimide. Immunoprecipitation was performed with 1 µg anti-FLAG M2 antibody (Sigma-Aldrich) for 4 h followed by incubation with protein A coupled sepharose beads for 1 h. Samples were washed excessively with lysis buffer, eluted with Laemmli buffer and subjected to Western blotting.

### Investigation of Smad/non-Smad signalling and Smad protein level

24 h post-transfection, C2C12 cells were starved in serum-free medium for 3 h and stimulated with 10 nM BMP2. At indicated times, cells were lysed in Laemmli buffer and subjected to Western blotting. To study protein turnover, cells were incubated with 5 µg/ml cycloheximide (Sigma-Aldrich) or EtOH as vehicle control applied in serum-free medium for 6 h. For inhibition of proteasomal activity, cells were incubated with 10 µM MG132 or DMSO as vehicle control applied in serum-free medium for 6 h.

### Differentiation Assay

C2C12 cells were transfected in suspension and seeded at high density; immortalised human myoblasts were seeded and transfected with siRNA as described. At confluency, differentiation was induced using DMEM (C2C12 cells) or OptiMEM (human myoblasts; Invitrogen) containing 2% horse serum (HS; PAA) in the absence or presence of 5 nM BMP2 or 10 nM BMP2, respectively. At indicated times, cells were harvested by addition of Laemmli buffer or RNA lysis buffer. Lysates were subjected to Western blotting or quantitative real-time PCR.

### *In situ* proximity ligation assay

Immortalised human myoblasts seeded in Nunc Lab-Tec II 16-well glass chamber slides (Thermo Scientific) were subjected to Duolink *in situ* proximity ligation (Sigma-Aldrich) as previously described^77^ using the following antibodies: BMPRII (#612292, BD Biosciences), IRS4 EP907Y (#TA303856, Origene), Smad1 (ab55476, Abcam), Smad4 (#9515, Cell Signaling).

### Immunofluorescence staining

C2C12 cells were transfected in suspension and seeded at high density. At confluency, differentiation was induced using DMEM containing 2% HS in the absence or presence of 5 nM BMP2. After 3 days, cells were fixed with 4% paraformaldehyde, quenched in 50 mM ammonium chloride, permeabilised with 0.2% Triton X-100 in 1x PBS and blocked in 2% BSA/1% FCS. Cells were stained using α-myosin heavy chain (Sigma-Aldrich) and Alexa Fluor^®^ 488 goat-α-mouse IgG (#A11001, Invitrogen) antibodies. For the staining of differentiated primary foetal myoblasts or satellite cells, α-MF20 (DSHB, #AB_2147781) and Alexa Fluor^®^ 488 goat-α-mouse IgG or Phalloidin594 Conjugate (sc-363795, Santa Cruz) were used. Nuclei were stained using DAPI (Sigma-Aldrich). Images were acquired by epifluorescence microscopy (Zeiss Axiovert 200), processed using AxioVision software and quantified using ImageJ by creating a threshold mask for the MHC channel with subsequent size gating; the MHC positive area was compared to the sum image area.

Primary myoblasts derived from E18.5 foetuses or primary postnatal satellite cells were seeded on glass cover slips, fixed with 4% PFA and blocked with TSA puffer containing 1x PBS, 10% HS, 0.5% (w/v) blocking reagent (Perkin Elmer) and 0.1% Triton X-100. Cells were stained using an α-IRS4 antibody (Bioss), α-MyoD antibody (BD Biosciences; #554130) or α-Pax7 antibody (DSHB, #AB_528428) and Alexa Fluor 568 goat-α- rabbit or Alexa Fluor 488 donkey –α-mouse antibodies (#A11011; A21202, Invitrogen). Nuclei were stained with DAPI. Images were acquired by confocal microscopy (Zeiss LSM700) and processed using AxioVision software.

### Flow cytometry

C2C12 cells were transfected as described. Where indicated, cells were starved in serum-free DMEM for 3 hours and stimulated with 10 nM BMP2 24 h post-transfection. Cells were harvested and fixed/permeabilised by incubation in ice cold 100% EtOH overnight at −20°C. After blocking in 1% BSA/1x PBS cells were co-stained with anti-myc tag (Cell Signaling), anti-Smad1 (Cell Signaling) or anti-Smad5 (Proteintech) antibodies recognized by R-phycoerythrin goat-α-mouse (#P852, Invitrogen) and Alexa Fluor^®^488 goat-α-rabbit IgG (#A11034, Invitrogen) antibodies. Immortalised human myoblasts were differentiated at confluence using OptiMEM containing 2% HS in the absence or presence of 10 nM BMP2. Cells were harvested and fixed/permeabilised using EtOH. Cells were stained using an α-IRS4 antibody (Origene) recognised by Alexa Fluor^®^488 goat-α-rabbit IgG antibody (#A11034, Invitrogen); as control nonspecific isotype control IgG (#3900, Cell Signaling) was used. Measurements were performed using an Epics XL-MCL flow cytometer (Beckman-Coulter). Data were evaluated using FCS3.0 Express (Denovo Software).

### Dual Luciferase Reportergene Assay

Cells were either transfected with a BMP response element reporter construct (BRE-luc)^31^ or a reporter construct containing the promoter region of myogenin (Myg-luc)^37^ together with other expression constructs as indicated. A constitutively expressing construct encoding renilla luciferase (RL-TK; Promega) was co-transfected as internal control. The next day, cells were starved in serum-free medium for 3 h or with DMEM containing 0.5% FCS for 5 h and stimulated with BMP2 for 6 h or overnight. For the Myg-Luc assay, cells were transfected in suspension and myogenic differentiation was induced at confluency using DMEM containing 2% HS in the absence or presence of 5 nM BMP2; cells were incubated for 3 days. Cell lysis was performed using passive lysis buffer (Promega) and measurement of luciferase activity was carried out according to manufacturer’s instructions using a Mithras LB940 Luminometer (Berthold Detection Systems).

### Quantitative real-time PCR

Total RNA extraction was performed using NucleoSpin^®^ RNA II isolation kit (Machery&Nagel) according to manufacturer’s instructions; 1 µg of RNA was subjected to reverse transcription using MMLV reverse transcriptase (Promega) and NV-oligo-dT primers (Invitrogen). Gene expression was assessed by quantitative PCR utilising StepOne Plus and SYBR Green PCR Master Mix (Applied Biosystems). Transcript expression levels were calculated as mean normalised expression (MNE) ratios referred to *HPRT* as housekeeping gene using the ΔΔ_CT_ method considering primer efficiency correction^78, 79^. All measurements were done in triplicates and C_T_ values were determined with the StepOne Software version 2.2 (Applied Biosystems). Primer sequences and gene accessions numbers are depicted in Supplementary Table S2.

## Electronic supplementary material

Supplemental Information
