# Supplementary material for: IRS4, a novel modulator of BMP/Smad and Akt signalling during early muscle differentiation
Source: Sci Rep. 2017 Aug 18;7:8778. doi: 10.1038/s41598-017-08676-6 (PMC5562708; doi:10.1038/s41598-017-08676-6)
Supplement: Supplementary file 1 — Supplemental Information [file 41598_2017_8676_MOESM1_ESM.pdf]

Supplementary Information

IRS4, a novel modulator of BMP/Smad and Akt signalling during early muscle differentiation

Gina Dörpholz, Arunima Murgai, Jerome Jatzlau, Daniel Horbelt, Mohammad Poorgholi Belverdi, Christina Heroven, Isabelle Schreiber, Gisela Wendel, Karen Ruschke, Sigmar Stricker, and Petra Knaus

Supplementary Figures

Figure S1

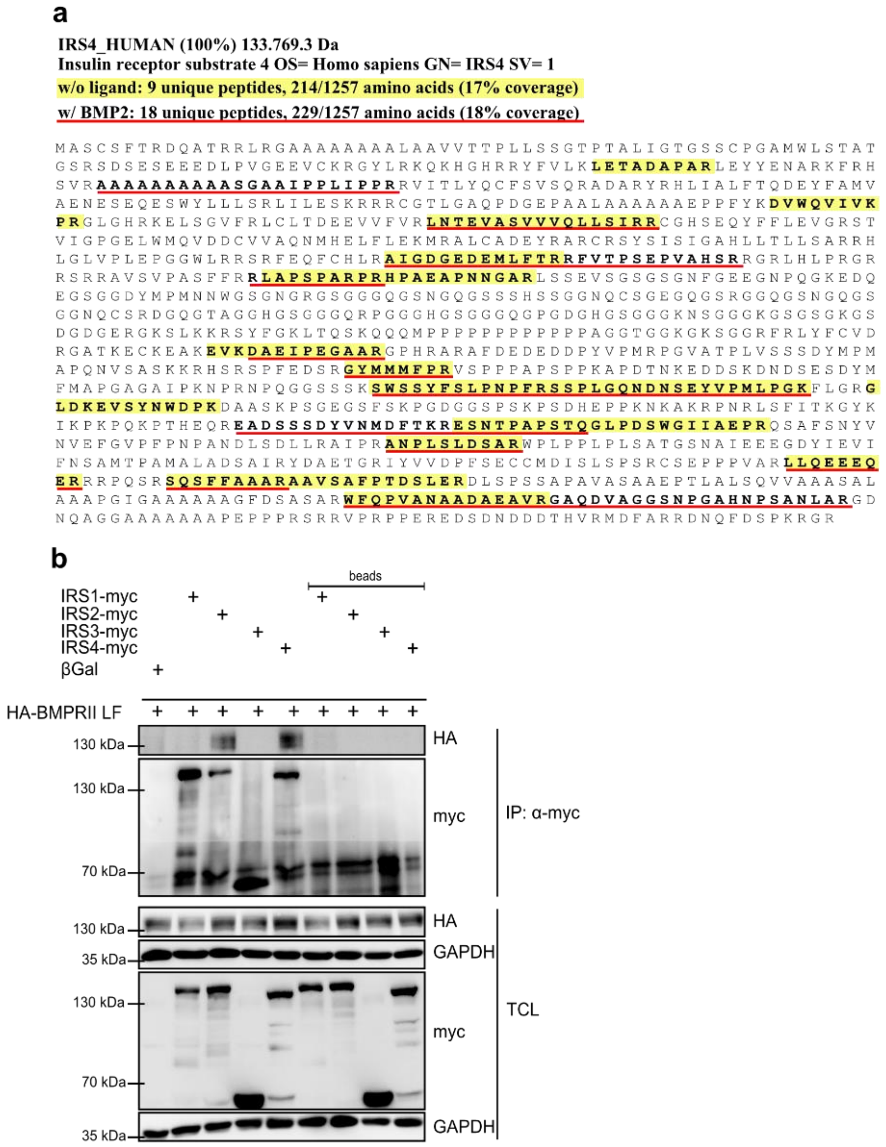

Supplementary Figure S1: IRS4 interacts with BMPRII

(a) Identification of IRS4 as a BMPRII interacting protein. IRS4 specific peptides were identified by mass spectrometry upon precipitation of HA-BMPRII-LF from HA-BMPRII stably transfected HEK293T cell lysates both in the absence (peptides marked in yellow) and presence of BMP2 (peptides underlined in red). **(b)** IRS4 and IRS2 are capable of interacting with BMPRII-LF. Transfected HEK293T cells were subjected to immunoprecipitation using an α-myc antibody; precipitates and TCL were analysed by Western blotting using indicated antibodies. Incubation with beads only served as IP control.

Figure S2

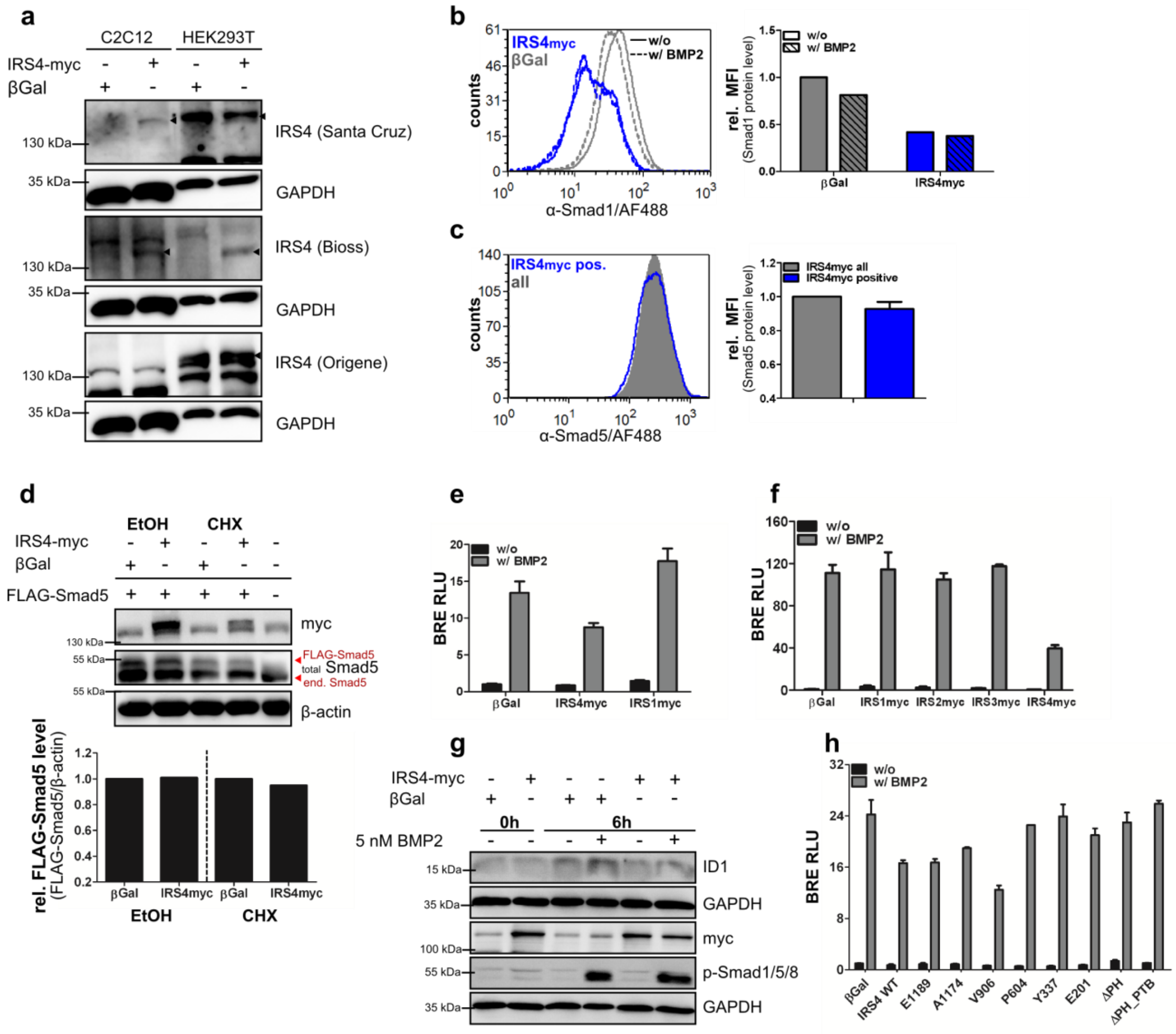

Supplementary Figure S2: Impact of IRS4 on Smad proteins and on transcriptional activity of Smads

(a) Expression of IRS4. HEK293T cells and C2C12 cells were transfected with IRS4-myc or β-galactosidase as control; lysates were subjected to Western blotting using indicated antibodies. The expected molecular weight of either overexpressed mouse IRS4 or endogenous protein are indicated by arrows. IRS4 Santa Cruz antibody detects particularly human IRS4, IRS4 Bioss and Origene antibodies detect solely the mouse or human protein, respectively. **(b)** The IRS4-induced decrease of Smad1 protein level is ligand-independent. Transfected C2C12 cells were stimulated with 10 nM BMP2 for 30 min and subjected to flow cytometry analysis. IRS4 transfected cells were gated on single cells (blue curve) and the endogenous Smad1 level was quantified. Solid lines depict unstimulated and dotted lines BMP2 stimulated samples. Bar chart shows MFI values relative to β-galactosidase w/o ligand. **(c)** IRS4 does not affect Smad5 protein levels. Transfected C2C12 cells were gated on myc-positive single cells (blue curve) via flow cytometry and the endogenous Smad5 level was analysed. Bar chart depicts MFI values of myc-positive cells compared to those of all cells (grey curve) derived from 2 independent experiments. **(d)** IRS4 does not affect FLAG-Smad5 levels**.** Transfected C2C12 cells were incubated with 5 µg/ml cycloheximide for 6 h; ethanol

was applied as vehicle control. Lysate were subjected to Western blotting using indicated antibodies. Quantification depicts total FLAG-Smad5 levels (see red indication on the blot) normalised to β-actin relative to β-galactosidase. **(e) (f)** Impact of IRS proteins on transcriptional activity of BMP Smads. C2C12 cells transfected with BRE-luc, RL-TK and IRS1-4-myc or β-galactosidase were stimulated with 5 nM BMP2 for 6 h (a) or 24 h (b), respectively. Bar chart depicts means ± SD of RLU from triplicate measurements relative to β-galactosidase. **(g)** IRS4 reduces BMP2-induced ID1 protein expression. Transfected C2C12 cells were stimulated with 5 nM BMP2 for 6 h. Lysates were subjected to Western blotting using indicated antibodies. **(h)** C2C12 cells transfected with BRE-luc, RL-TK and IRS4-myc, truncations thereof or β-galactosidase were stimulated with 5 nM BMP2 for 6 h. Bar chart depicts means ± SD of RLU from triplicate measurements relative to β-galactosidase. RLU= relative luciferase units (BRE-luc/RLTK-luc)

Figure S3

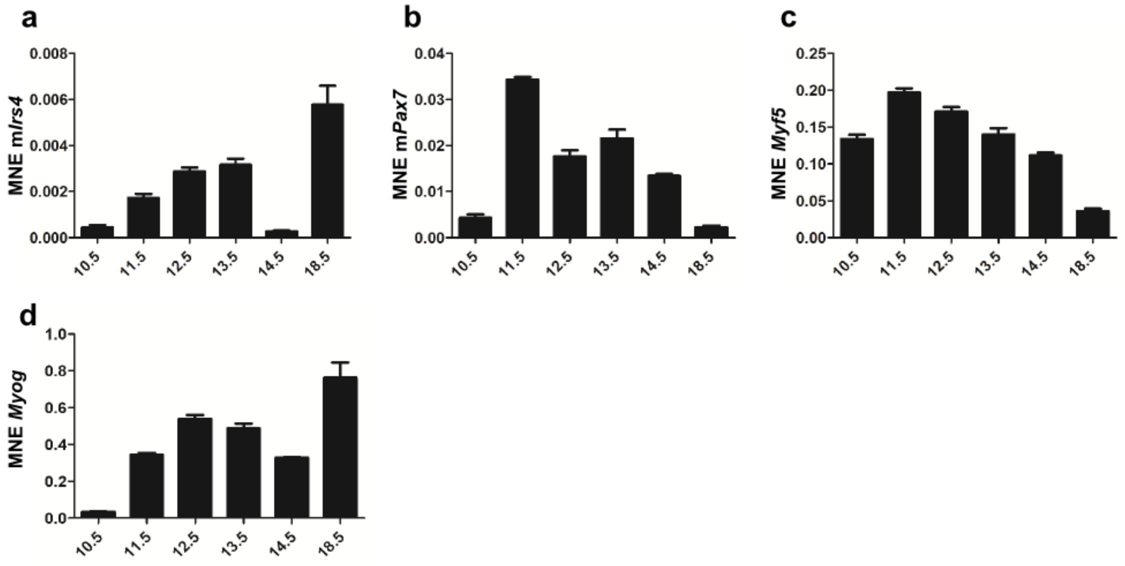

Supplementary Figure S3: Expression of IRS4 and myogenic markers during mouse limb development

(a-d) IRS4 is expressed during mouse limb development. RNA from mouse limbs of indicated embryonic developmental stages was isolated, reverse-transcribed and subjected to gene expression analysis via qRT-PCR. Bar charts summarise triplicate measurements and depict MNE ± SEM representative for 2 independent experiments. Data corresponds to these combined in Fig 5a.

Figure S4

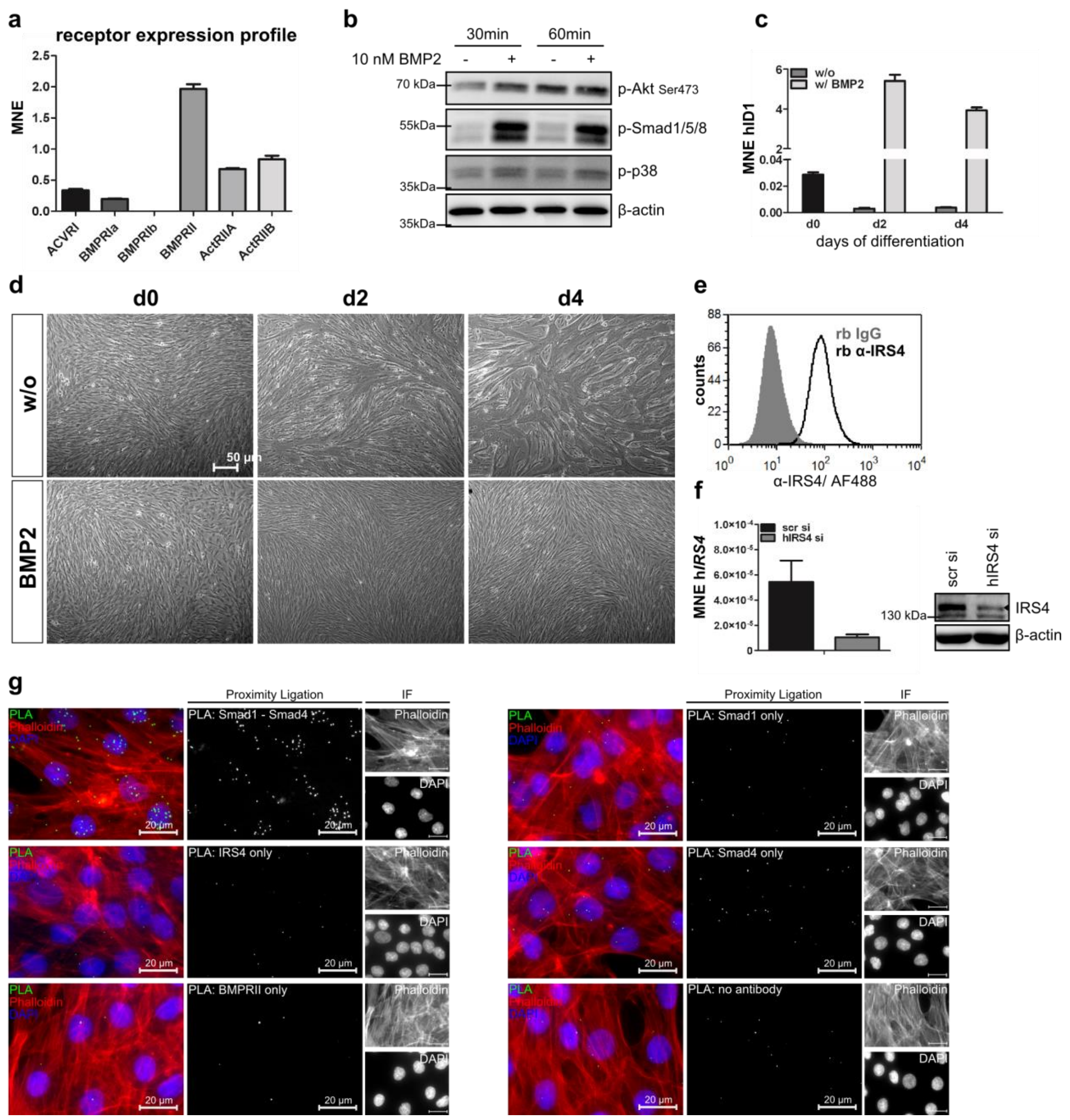

Supplementary Figure S4: Characterisation of BMP signalling in immortalised human myoblasts

(a) RNA from cultured human myoblasts was isolated, reverse-transcribed and used for gene expression analysis via qRT-PCR. Bar chart depicts means ± SEM from triplicate measurements. **(b)** Human myoblasts were stimulated with 10 nM BMP2 for indicated times; lysates were subjected to Western blotting using indicated antibodies. **(c)** Human myoblasts were differentiated with or without 10 nM BMP2 for indicated times; gene expression was analysed via qRT-PCR. Bar chart depicts MNE ± SEM from triplicate measurements. **(d)** Concomitantly, images were acquired at indicated times. **(e)** IRS4 is expressed in human myoblasts. IRS4 expression was verified via flow cytometry using a specific antibody (black line; control IgG: grey shaded curve). **(f)** siRNA-mediated knockdown of IRS4. Knockdown efficiency was analysed via qRT PCR (left) and Western blotting (right) **(g)** Control samples for the *In situ* proximity ligation assay (PLA) of IRS4 and BMPRII or Smad1. Human myoblasts

were subjected to *in situ* PLA (green signal) to visualize the endogenous association of IRS4 with BMPRII or Smad1; nuclei and the actin cytoskeleton were stained using DAPI and Phalloidin594. The association of Smad1 with Smad4 served as positive control.

Figure S5

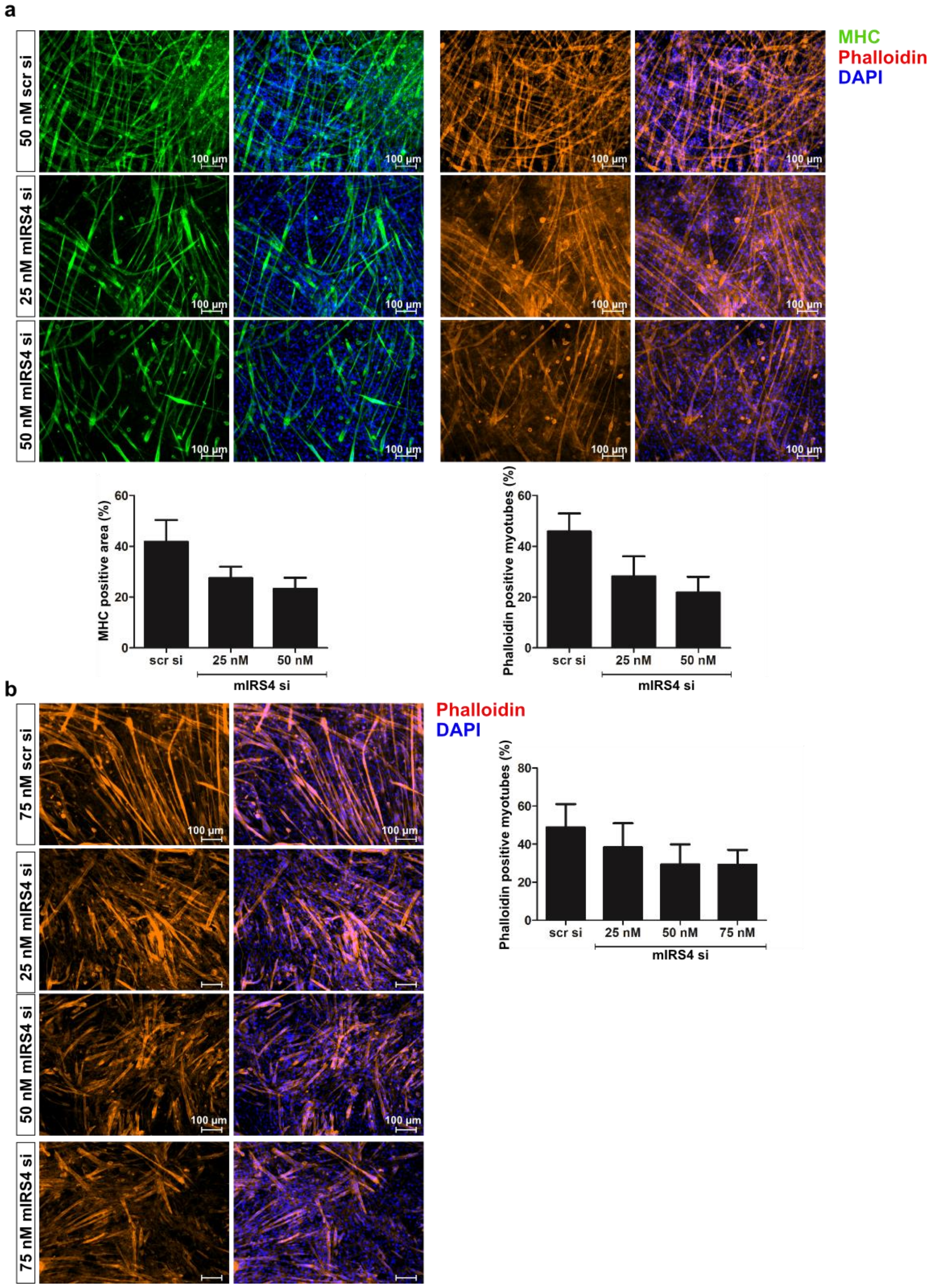

Supplementary Figure S5: Targeted IRS4 knockdown decreases myogenic differentiation of primary foetal myoblasts and primary postnatal satellite cells
(a) (b) IRS4 knockdown affects myogenesis in primary mouse cells. Primary mouse myoblasts isolated from E18.5 limb muscles (a) or primary mouse satellite cells isolated from P7 limb muscles (b) were transfected with siRNA targeting either nonspecific sequences (scr si) or mouse IRS4 (mIRS4 si) and differentiated for three (a) or two (b) days, respectively. Myosin heavy chain (MHC) and/or the actin cytoskeleton were stained using a specific antibody or Phalloidin594; nuclei were stained using DAPI. Images were quantified using ImageJ. Bar charts depict means ± SD of at least 16 images per condition.

Supplementary Materials and Methods

Supplementary Table S1: Primers for cloning

Primer name	sequence 5'-3'
mIRS1 fwd	CCACCATGGCGAGCCCTCCGG
mIRS2 fwd	CCACCATGGCTAGCGCGCCCC
rIRS3 fwd	CCACCATGAAGCCTGCAGGTACGG
mIRS4 fwd	CCACCATGGCGAGTTGCTCCTTCT
Myc c-term rev	ATGAAGATGAGGATGACCCG
rIRS3 rev	TAGCTATGCCAGCATCAAGTTCGGAGGTGAACAAAAGCTAATATCAGAAGAAGATCTTTAA
mIRS4 myc rev	AAGCTTATTTGAGGAAGATCTTTAG
mIRS4 E201stop fwd	TCAGCCGCCTCATCCTCTAAAGCAAGCGCCGCCGCTG
mIRS4 E201 myc stop rev	AGCCGCCTCATCCTCGAGGGAGGTGAACAAAAGCTTATTTCTGAGGAAGACCTTTAC
mIRS4 Y337stop fwd	AGCCTTGTGCGCAGATGAATGGAGAGCCCGATGCCGCAGCTAC
mIRS4 Y337myc stop rev	GCCTTGTGCGCAGATGAATACGGAGGTGAACAAAAGCTTATTTCTGAGGAAGACCTTTAG
mIRS4 P604stop fwd	AGCAGCAGCAAACACTGTGACCACCCCCACCGCCACC
mIRS4 P604 myc stop rev	GCAGCAGCAAACACTGCCTGGAGGTGAACAAAAGCTTATTTCTGAGGAAGACTTTTAG
mIRS4 P908stop fwd	AGAGAGCGCCTTGTGCTATGATCCTCTGCTCAAGGACTG
mIRS4 V906 myc rev	ATTAAGAGAGAGCGCCTTGTGGGTGGAGAGCAGAAACTAATCTCTGAAGAGGACCTATAA
mIRS4 P1175stop fwd	GCCGGCGCAGCGGCTTAACCGCCGCCACCTCGC
mIRS4 A1174 myc rev	CGGCGCAGCGGCTGGTGGAGAGCAAAAACTAATCTCTGAGGAAGACCTATAA
mIRS4 E1189stopfwd	TGGGTGCTGAGACCCCAATAGAGAGCGGATTCTGAGG
mIRS4 E1189myc rev	GGTGCTGAGACCCCAAGAGGGTGGAGAGCAGAAACTAATCTCTGAAGAGGACCTATAA
mIRS4 dPH fwd	CCACCATGGAGAGCAAGCGCCG
mIRS4 dPH PTB fwd	CCACCATGTACAGAGCCCGATGCC

Supplementary Table S2: Primers for qRT-PCR

primer	species	sequence 5'-3'	accession number
IRS4	Human	**F** TCTTGCTGACAGTGCCATTC<br>**R** CTTTGTGGGCGTCTTCTCTC	ENST00000372129<br>NM_003604.2
ID1	Human	**F** GCTGCTCTACGACATGAACG<br>**R** CCAACTGAAGGTCCCTGATG	NM_002165
HPRT	Human	**F** CTTTGCTGACCTGCTGGATT<br>**R** CTGCATTGTTTTGCCAGTGT	NM_000194
MyoG	Human	**F** CAGGGGTGCCCAGCGAATGC<br>**R** ATCTGTAGGGTCAGCCGTGA	ENST00000241651<br>NM_002479.5
ACVRI	Human	**F** AAGCCTGGAGCATTGGTAA<br>**R** TCACTGGGGTACTCGGAGA	NM_001105
BMPRIa	Human	**F** CATCTTGGAGGAGTCGTAAGAA<br>**F** TTCTGTCCTTGAACACGAGAAA	NM_004329
BMPRIb	human	**F** CTGCCATAAGTGAGAAGCAAAC<br>**R** ACAACGCAAGACCTTTGGAC	NM_001203
ActRIIA/ACVR2A	Human	**F** CCTGACAGCTTGCATTGCTGACTT<br>**R** TCTGCGTCGTGATCCCAACATTCT	NM_001616
ActRIIB/ACVR2B	Human	**F** TGAAGCACGAGAACCTGCTACAGT<br>**R** GGCATACATGTCAATGCGCAGGAA	NM_001106
BMPRII	Human	**F** CATGGAGATGCGTAGCTGTC<br>**R** GGTTCTGAGGAAGTGCGAGT	NM_001204
IRS4	Mouse	**F** GTGCTGAGACCCCAAGAGAG<br>**R** TGGTTTTGGTGGCAGTGTAA	NM_010572.2
ID1	Mouse	**F** CTTCAGGAGGCAAGAGGAAA<br>**R** CAAACCCTCTACCCACTGGA	NM_010495
Pax7_1	Mouse	**F** CTGGATGAGGGCTCAGATGT<br>**R** GGTTAGCTCCTGCCTGCTTA	NM_011039.2
Pax7_2	Mouse	**F** CCGTGTTTCTCATGGTTGTG<br>**R** GAGCACTCGGCTAATCGAAC	NM_011039
Myf5	Mouse	**F** CTGTCTGGTCCCGAAAGAAC<br>**R** GCTCGGATGGCTCTGTAGAC	NM_008656.5
MyoG_1	Mouse	**F** CCAAGGTCTCCTGTGCTGATG<br>**R** TTGGCAAAACCACACAATGC	NM_031189.2
MyoG_2	Mouse	**F** CTACAGGCCTTGCTCAGCTC<br>**R** AGATTGTGGGCGTCTGTAGG	NM_031189
MyoD	Mouse	**F** AGCACTACAGTGGCGACTCA<br>**R** GCTCCACTATGCTGGACAGG	NM_010866
GAPDH	Mouse	**F** AACTTTGGCATTGTGGAAGG<br>**R** CAGTCTTCTGGGTGGCAGTG	NM_008084
HPRT_1	Mouse	**F** TGTTGTTGGATATGCCCTTG<br>**R** ACTGGCAACATCAACAGGACT	NM_013556
HPRT_2	Mouse	**F** TATGCCGAGGATTTGGAAAA<br>**R** TGTAATCCAGCAGGTCAGCA	NM_013556

Target sequences of used siRNAs

All siRNA oligonucleotides (ON-TARGET plus) were obtained from Thermo Scientific/Dharmacon.

siRNA hIRS4 (hIRS4 si) target sequence: GGG-AAG-AUU-CAG-ACU-UUA-U

siRNA mIRS4 (mIRS4 si) SMART pool, target sequences: UCA-AAC-AGG-UCA-AAU-CUA-U, CAG-CAU-AGG-CGACGUCUU-U, GAA-CUC-AAG-UAA-AGC-CCA-A, CAA-AGA-AGC-CAA-AGAGGU-A

Non-targeting siRNA#1 (scr si) target sequence: UGG-UUU-ACA-UGU-CGA-CUA-A

Antibodies

The following antibodies were used for Western blotting: β-actin (#A5441, Sigma-Aldrich), GAPDH (#2118, Cell Signaling), ID1 (sc-488, Santa Cruz), Caveolin-3 (sc-5310, Santa Cruz), myogenin (sc-13137), myosin [skeletal, fast] (#M4276, Sigma-Aldrich), total Smad1 (#6944, Cell Signaling), total Smad5 (#12167-1-1-AP, Proteintech) , p-Smad1/5/8 (#9511, Cell Signaling), p-Akt Ser473 (#4060S, Cell Signaling), p-GSK3β Ser9 (#9336, Cell Signaling), p-p70S6K Thr421/Ser424 (#9204, Cell Signaling), p-p38 pTGpY (#V1211, Promega), IRS4 (sc-28830, Santa Cruz; bs-0187R, Bioss; #TA303856, Origene), p-Tyrosine-1000 (#8954, Cell Signaling), BMPRII (#612292, BD Biosciences), FLAG® Tag M2 (#F3165, Sigma Aldrich), HA Tag (#H3663, Sigma-Aldrich) and myc Tag (#2276, Cell Signaling) followed by HRP-conjugated secondary antibodies goat-α-mouse/rabbit (#115-035-068/#111-035-046, Dianova).

Isolation of primary foetal mouse myoblasts and cytospin

For the isolation of primary myoblasts, limbs derived from E18.5 foetuses were used. First, the muscle tissue was dissected from the skin and bones and homogenised. Homogenates were transferred into Hank's buffered Salt Solution (HBSS; Biochrom AG/Thermo Scientific) supplemented with 2 mM $MgSO_4$ and 2 mM $CaCl_2$. Then, 50 µl 15% collagenase (Sigma-Aldrich) was added and incubated at 37°C and 1000 rpm for 40 min with rigorous vortexing in between. Each sample was supplemented with growth medium (DMEM (inc. 4,5 g/l glucose) BioWhittaker Cambrex), pipetted through a 40 µM cell strainer into 15 ml tubes and centrifuged at room temperature at 2000 rpm for 5 min. The cell pellet was resuspended in growth medium, added onto poly-D-lysine (1 mg/ml; Millipore) coated glass cover slips, incubated for 1 h at

room temperature and subjected to cytospin-preparation at 200 rpm for 5 min followed by fixation and immunofluorescence staining. For RNA isolation, cDNA synthesis and semi-quantitative PCR analysis, pure primary myoblast population was used.

Isolation of primary postnatal mouse satellite cells

Forelimb and hindlimb muscles harvested from day 7 postnatal mice (P7) were minced and digested using 20 µl collagenase A (100 µg/µl; Sigma-Aldrich) in DMEM in a shaking water bath at 37°C and 1400 rpm for 30 min followed by addition of 2 U/ml dispase (Sigma-Aldrich) for another 30 min. Next, the sample was passed through a 20G syringe followed by a 70 µm cell strainer to obtain a single cell suspension.

For the isolation of satellite cells via fluorescence-activated cell sorting (FACS), fluorescently labelled antibodies as listed below were added to the single cell suspension and incubated for 1 h on ice. The cells were washed three times in HBSS by centrifugation at 4°C 300 g for 5 min and subsequently resuspended in 500 µl HBSS containing 5% BSA. Cells were sorted via FACS-based exclusion to deplete cells expressing surface markers CD45, CD31, Ly-6A/E-Sca1 and Ter119 and positive selection for α-7 integrin using a BD FACSAria II (BD Biosciences).

Supplementary Table S3: Antibodies used for FACS-based sorting of primary mouse satellite cells

Antigen	Fluorophore	Used amount	provider	Ordering number
CD45	APC	10 µl	Life Technologies	#17-0451-83
CD31	APC	10 µl	Life Technologies	#17-0311-82
Ly-6A/E-Sca1	APC/Cy7	10 µl	Biolegend	#108126
Ter119	APC	10 µl	Life Technologies	#17-5921-83
α-7 integrin	PE	5 µl	AbLab	#53-0010-05

Cultivation and transfection of primary mouse myoblasts and satellite cells

Sorted satellite cells were either seeded on matrigel coated (Corning® Matrigel® Basement Membrane Matrix Growth Factor Reduced) 10-mm cover slips for immunofluorescence stainings or 24-well plates for RNA isolation, cDNA synthesis and subsequent semi-

quantitative PCR analysis. Cells were cultured in DMEM containing 20% FCS, 10% horse serum, 2 mM L-glutamine and penicillin (100 units/ml)/streptomycin (10 µg/ml) at 37°C and 5% CO_2; the medium was exchanged every other day.

Differentiation was induced using DMEM supplemented with 5% horse serum, 2 mM L-glutamine and penicillin (100 units/ml)/streptomycin (10 µg/ml).

For siRNA-mediated knockdown of mouse IRS4 in primary myoblasts or satellite cells, Lipofectamine RNAiMAX (Invitrogen) was used according to manufacturer's instructions; as control non-targeting siRNA was used. In short, cells were transfected with 25-75 nM siRNA and differentiation was induced 48 h post-transfection.

Semi-quantitative PCR analysis

RNA isolated from freshly isolated, pure primary myoblasts or isolated and cultured primary satellite cells was subjected to cDNA synthesis and amplified by PCR using 2 µl cDNA template, 2 µl dNTPs (1.25 mM; Fermentas), 1 µl of each specific primer (10 µM, see Supplementary Table S2) and 0.5 µl Taq polymerase (5-10 U/µl; homemade) in a final reaction volume of 20 µl. The amplification reaction consisted of an initial denaturation step at 95°C for 10 min followed by 30 cycles of 15 sec at 95°C and 60 sec at 60°C. PCR products were separated on 1.5% agarose gels and visualised via ethidium bromide staining.
